# Combined *Bacillus velezensis*–*Brevibacterium frigoritolerans* Biostimulant and Melatonin Improves Salt Tolerance in Maize Seedlings and Is associated with Physiological and Transcriptome–Metabolome Changes

**DOI:** 10.4014/jmb.2606.06019

**Published:** 2026-10-13

**Authors:** Xiaoyong Li, Chen Wang, Qing Feng, Yu Qiao, Yuning Zhang

**Affiliations:** College of Environmental Science & Engineering, Qilu University of Technology (Shandong Academy of Sciences), Jinan, Shandong 250353, P. R. China

**Keywords:** *Bacillus velezensis*, *Brevibacterium frigoritolerans*, Integrated omicsm, Microbial biostimulant, Salinity stress

## Abstract

Salt stress severely limits maize seedling establishment, and microbiology-based biostimulants offer a promising strategy for improving crop performance under salinity. However, whether plant growth-promoting rhizobacteria (PGPR) can complement melatonin (MT) in regulating maize salt responses remains unclear. Here, maize seedlings grown hydroponically under 100 mM NaCl were treated with a mixed PGPR inoculant comprising *Bacillus velezensis* and *Brevibacterium frigoritolerans*, foliar MT, or their combination. Physiological traits were measured across eight treatments, and comparative transcriptomic and metabolomic analyses were performed between salt stress alone (S) and the combined treatment (S+R+MT). The combined treatment more effectively restored plant height, root length, biomass, root activity, photosynthetic pigments, proline, soluble sugars, and antioxidant enzyme activities than single treatments, while decreasing malondialdehyde and H_2_O_2_ accumulation. Untargeted metabolomics identified 836 differential metabolites, mainly enriched in amino acid biosynthesis and metabolism, the pentose phosphate pathway, nucleotide metabolism, and ABC transporters. Transcriptome analysis identified 7,307 differentially expressed genes enriched in carbon metabolism, amino acid biosynthesis, starch and sucrose metabolism, phenylpropanoid biosynthesis, and MAPK signaling. Integrated analysis indicated that the combined treatment coordinated osmotic adjustment, carbon allocation, glutathione/ascorbate-associated redox buffering, and phenylpropanoid-related defense. These results indicate that co-application of PGPR and MT can improve the salt tolerance of maize seedlings under controlled hydroponic NaCl stress.

## Introduction

Maize (*Zea mays* L.) is a major crop for food, feed, and industrial use, and its stable and high productivity is of great significance for ensuring food security[1]. In China, saline-alkali land covers nearly 100 million hectares, accounting for roughly 10.1% of the worldwide total, and its expansion has been linked to irrigation mismanagement, groundwater mineralization, and climate change [2]. Among the various stresses associated with saline environments, salt-induced osmotic and ionic stresses caused by excessive soluble salts represent major factors restricting maize growth and development.

The effects of salt stress on plants vary with developmental stage. The initial effect is osmotic stress resulting from reduced soil water potential, which rapidly inhibits leaf expansion and plant growth. This is followed by ionic toxicity caused by excessive accumulation of Na^+^ and Cl^−^, further leading to premature leaf senescence, impaired photosynthesis, and yield loss [3]. Salt stress also promotes excessive ROS production, which can trigger membrane lipid peroxidation, pigment loss, and metabolic disruption, thereby inhibiting plant growth [4, 5]. Maize is a typical salt-sensitive cereal crop, especially during the germination and seedling stages[6]. Previous studies have shown that salt stress reduces leaf relative water content, net photosynthetic rate, and chlorophyll content [7]. It also promotes lipid peroxidation and disrupts antioxidant defense, thereby affecting biomass accumulation and overall plant growth[8]. Because germination and seedling establishment are critical for subsequent stand establishment and productivity in maize, clarifying salt-tolerance regulation at the seedling stage is of practical importance [9].

In addition to growth inhibition, salt stress also disturbs central cellular metabolism. Under salinity, carbon flux is often shifted away from growth and toward osmotic adjustment and antioxidant defense, while nitrogen metabolism is redirected to support the synthesis of amino acids and other stress-related compounds [3]. Therefore, evaluating salt tolerance only from the perspective of morphology or individual physiological indices is often insufficient, and the integration of physiological measurements with transcriptomic and metabolomic evidence is increasingly considered necessary for a more comprehensive understanding of plant salt adaptation.

The improvement of crop salt tolerance currently relies mainly on three approaches: selection of salt-tolerant varieties, genetic improvement, and exogenous regulation [10]. Although breeding and genetic improvement have long-term value, salt tolerance is governed by multiple genes and involves ion transport, osmotic adjustment, ROS scavenging, antioxidant defense, and signal transduction [11], making rapid improvement through conventional breeding alone difficult. In contrast, exogenous regulatory approaches have attracted extensive attention in studies of crop stress tolerance because they are considered practical short-term interventions with relatively rapid physiological effects [12].

Melatonin (MT) is an indole small molecule widely present in plants and possesses strong antioxidant and stress-regulatory functions. Exogenous melatonin has been widely reported to improve plant stress tolerance by limiting ROS buildup, strengthening antioxidant defense, reducing membrane lipid damage, and modulating stress-related gene expression [13]. Chen *et al*. [14] reported that 100 μM exogenous MT could alleviate salt stress-induced ROS accumulation and improve photosynthetic efficiency. Nevertheless, the regulatory effects of MT are often characterized by rapid responses [15], whereas long-term stress adaptation may also depend on rhizosphere-mediated and root-associated regulation. Plant growth-promoting rhizobacteria (PGPR) can help plants cope with stress by stimulating root growth, improving water and nutrient uptake, maintaining phytohormone balance, and strengthening antioxidant defense [16]. The positive effects of PGPR largely depend on rhizosphere activity, but their effectiveness can vary with environmental conditions, colonization success, and the stability of plant–microbe interactions [17].

From an applied microbiology and biotechnology perspective, PGPR represent functional microbial resources that can be developed as crop biostimulants. Unlike direct chemical amendments, PGPR-based treatments act through root-associated biological processes, including rhizosphere activity, root physiological stimulation, and stress-responsive plant–microbe signaling. For saline environments, however, the practical value of a microbial inoculant depends on whether the bacteria remain active in the root-associated fraction and whether this microbial activity translates into measurable plant stress protection. Therefore, evaluation of PGPR-based salt-alleviation strategies should combine plant physiological responses with evidence for microbial persistence and downstream molecular regulation.

From a mechanistic perspective, MT and PGPR may complement each other at multiple levels. MT primarily acts within plant tissues as a rapid regulator of redox status, membrane stability, photosynthesis, and stress-responsive signaling [14], whereas PGPR mainly functions through root-associated processes, including improvement of nutrient and water acquisition, modulation of phytohormone balance, and stimulation of systemic stress defense [18]. These complementary roles suggest that co-application of PGPR and MT may enhance salt tolerance by linking aboveground physiological protection with belowground rhizosphere support [19], thereby jointly reshaping plant salt-stress responses across multiple functional levels.

In maize, most studies have examined MT or PGPR separately, and evidence for their combined effect remains limited. Existing work has also focused mainly on growth recovery or individual physiological traits, with less attention to how PGPR–MT co-application reshapes stress-related metabolism and gene expression [20]. Therefore, this study evaluated whether a defined PGPR consortium combined with MT could provide stronger protection against salt stress than single treatments in maize seedlings. It should be noted that this study investigated NaCl-induced salinity stress rather than saline-alkali stress. Further studies using soil-based saline-alkali models are needed to validate the broader applicability of PGPR–MT treatment. Physiological, biochemical, transcriptomic, and metabolomic analyses were integrated to link phenotype with stress-response pathways, and root-associated culturable bacterial populations were estimated to support interpretation of microbial persistence in the hydroponic system.

## Materials and Methods

### Plant Materials and Growth Conditions

Maize seedlings were cultivated hydroponically in a controlled greenhouse. Uniform and healthy seeds of the *Zea mays* L. cv. Zhengdan 958 were selected, sterilized with sodium hypochlorite for 15 min, and rinsed thoroughly with deionized water. The sterilized seeds were wrapped in wet gauze, placed in Petri dishes, and allowed to germinate in darkness at 25°C for 48 h. After germination, uniform seedlings were transferred to hydroponic boxes containing half-strength Hoagland nutrient solution. Each box had a volume of 3 L and contained 16 seedlings. The growth regime was maintained at a 16 h / 8 h light/dark cycle, with temperatures of 25°C during the light period and 20°C during the dark period, and a relative humidity of 60%.

The bacterial strains used in this study were *Brevibacterium frigoritolerans* and *Bacillus velezensis*, both of which were screened and obtained by our research group [21]. The strains were maintained in the laboratory culture collection and used here as a defined two-strain PGPR inoculant rather than an undefined rhizosphere community. A summary of their key plant growth-promoting traits, previously characterized under salt stress conditions, is provided in Table 1. All trait data were characterized under salt stress conditions (0–10% NaCl) and reported by Wang *et al*.[21]. “+” indicates positive activity; “++” indicates strong activity with enhancement under salt stress; “−” indicates no detected activity; “±” indicates activity detected only under low salinity (0–4% NaCl). These traits were used for preliminary characterization of the selected strains, whereas their contribution to plant responses was interpreted according to the experimental conditions.

### Experimental Design and Treatments

A preliminary test was performed to identify an appropriate melatonin (MT) concentration for maize seedling growth under salt stress. Five MT levels (0, 50, 100, 150, and 200 μM) were evaluated under 100 mM NaCl, and 100 μM MT was used in the subsequent experiment because it showed the strongest growth-promoting effect (Fig. S1–S2).

After 3 d of pre-culture in 1/2 Hoagland nutrient solution, uniformly growing maize seedlings with similar growth status were randomly assigned to eight treatments: CK, CK+R, CK+MT, CK+R+MT, S, S+R, S+MT, and S+R+MT. This setup enabled evaluation of the separate and combined effects of PGPR and MT under both control and salt-stress conditions.

For PGPR application, *Brevibacterium frigoritolerans* and *Bacillus velezensis* were separately cultured in LB liquid medium at 30°C and 180 rpm for 24 h. Cells were collected by centrifugation, washed twice with sterile saline to remove residual LB medium, and resuspended in sterile saline [22]. The final bacterial density in the hydroponic system was adjusted to 1 × 10^6^ CFU mL^−1^, representing the total density of the mixed suspension. For MT treatment, melatonin was dissolved directly in sterile distilled water to prepare a 100 μM solution. The MT solution was uniformly sprayed onto leaves every 3 d under light-avoidance conditions until the leaf surfaces were fully wetted without visible dripping. All non-MT treatments, including CK, CK+R, S, and S+R groups, received the same volume of sterile water spray using the identical procedure to exclude potential effects caused by foliar spraying. Salt stress was imposed by adding NaCl to the nutrient solution to a final concentration of 100 mM. Once the target salinity was established, seedlings were maintained under the respective treatment conditions for 14 d. During the treatment period, the nutrient solution was renewed every 7 d. For PGPR-treated groups, the bacterial suspension was re-inoculated after each renewal to maintain microbial activity in the hydroponic system. Continuous aeration was applied throughout the experiment to maintain root-zone oxygen supply. Samples for physiological, biochemical, metabolomic, and transcriptomic analyses were collected from independent biological samples at the end of the 14-d treatment period.Each treatment included three biological replicates, and each biological replicate consisted of one independent hydroponic box containing 16 maize seedlings. In this study, the hydroponic box rather than the individual seedling was regarded as the experimental unit. At the end of the treatment period, independent seedlings were used for different destructive analyses. One randomly selected seedling from each hydroponic box was used for physiological and biochemical measurements. Separate matched biological replicates from the same experimental batch were collected for transcriptomic and metabolomic analyses. Therefore, physiological measurements and omics analyses were performed using independent but corresponding biological replicates. Untargeted metabolomic and transcriptomic analyses were performed only for the S and S+R+MT groups in order to characterize the molecular response associated with the superior salt-alleviating effect of the combined treatment relative to salt stress alone.

### Determination of Root-Associated Culturable Bacterial Populations

At the end of the 14-d treatment period, root samples were collected from the S, S+R, and S+R+MT groups. Whole root systems of maize seedlings were carefully removed from the hydroponic solution, and loosely adhering solution was allowed to drain. The roots were then transferred to sterile 50 mL tubes and gently washed three times with sterile phosphate-buffered saline (PBS, pH 7.0) to remove non-adhering bacteria. After washing, root samples were blotted dry on sterile filter paper and weighed to record fresh root weight.

The washed roots were transferred to sterile mortar and homogenized thoroughly in 10 mL of sterile PBS. The homogenate was serially diluted in sterile PBS, and 100 μL aliquots from each dilution were spread-plated onto LB agar plates in triplicate. The plates were incubated at 30°C for 24 h. After incubation, colonies were counted, and the number of colony-forming units per gram of fresh root weight (CFU g^−1^ fresh root) was calculated. The enumeration was performed for three biological replicates per treatment. Because LB agar was non-selective, the assay estimated total culturable root-associated bacteria rather than strain-specific colonization.

### Measurement of Experimental Parameters

**Growth measurement.** At the end of the experimental period, one seedling from each biological replicate was randomly selected for measurement of plant height, root length, fresh weight, and dry weight. Root activity was evaluated using the triphenyl tetrazolium chloride (TTC) reduction assay [23].

**Determination of photosynthetic pigment contents.** Chlorophyll and carotenoid contents were measured with reference to a previously reported method [24]. Briefly, 100 mg of leaf tissue was finely ground in a cooled mortar, and the pigments were extracted with 80% acetone. The extract was centrifuged at 12,000 × g for 10 min, after which the absorbance of the supernatant was measured at the corresponding wavelengths. The contents of chlorophyll a, chlorophyll b, total chlorophyll, and carotenoids were then calculated. The relevant equations and detection wavelengths are given below.

$$C_{a}=12.21A_{663}-2.81A_{646}$$

$$C_{b}=20.13A_{646}-5.03A_{663}$$

$$\mathrm{Chl}=C_{a}+C_{b}$$

$$C_{x+c}=\frac{1000A_{470}-1.82C_{a}-85.02C_{b}}{198}$$

*C_a_*: Chlorophyll a; *C_b_*: Chlorophyll b; Chl: Total Chlorophyll; *C_x+c_*: Carotenoids

**Determination of osmotic adjustment substances.** The determination of osmotic adjustment substances was conducted with reference to previously reported methods [25]. Proline (Pro) was quantified using the acidic ninhydrin colorimetric method, whereas soluble sugar content was determined using the anthrone colorimetric method.

**Determination of MDA and H_2_O_2_ contents.** Malondialdehyde (MDA) content was assayed by the thiobarbituric acid (TBA) assay. Absorbance values at 532 and 600 nm were recorded, and the extent of lipid peroxidation in leaves was estimated accordingly [26]. Hydrogen peroxide (H_2_O_2_) content was analyzed using the KI assay [27]. Briefly, 100 mg of fresh leaf tissue was extracted with 5 mL of 0.1% trichloroacetic acid (TCA) and then centrifuged at 12,000 × g for 10 min. Then, 0.5 mL of the supernatant was combined with an equal volume of potassium phosphate buffer (pH 7.0), followed by addition of 1 mL KI solution. After the reaction was completed, absorbance was read at 390 nm.

**Determination of antioxidant enzyme activities.** Fresh maize leaf tissue (0.5 g) was extracted with 5 mL of 0.1 M phosphate buffer under ice-cold conditions. The extract was centrifuged at 12,000 × g for 15 min at 4°C, and the supernatant was collected for enzyme activity determination. POD activity was determined by the guaiacol assay, SOD activity by the nitro blue tetrazolium (NBT) assay, CAT activity by monitoring the decomposition of H_2_O_2_ in the reaction system, and APX activity by the ascorbate oxidation assay [28].

**Untargeted metabolomics analysis.** Leaf samples from the S and S+R+MT groups were collected at the end of the 14-d treatment period, immediately frozen in liquid nitrogen, and stored at -80°C until analysis. Frozen leaf tissues were ground in liquid nitrogen, and approximately 5 mg of powdered tissue was transferred into a 2 mL centrifuge tube. Metabolites were extracted with pre-cooled 80% methanol containing internal standards. The mixture was homogenized thoroughly, vortexed for 1 min, and centrifuged at 14,000 × g at 4°C for 10 min. The supernatant was collected, vacuum freeze-dried, and reconstituted in 100 μL of 10% methanol/90% water. After vortexing for 30 s, sonication for 1 min, and centrifugation at 14,000 × g at 4°C for 10 min, the supernatant was transferred to sample vials for LC–MS analysis. A pooled quality control sample was prepared by mixing equal aliquots of all sample extracts and was used to monitor the stability and reproducibility of the analytical process.

Metabolites were separated using a Vanquish Flex UPLC system equipped with a Waters ACQUITY BEH C18 column. The column temperature was maintained at 40°C, the autosampler temperature was set at 10°C, and the injection volume was 5 μL. Mobile phase A consisted of 0.1% formic acid in water, and mobile phase B consisted of 0.1% formic acid in acetonitrile/methanol. Mass spectrometric analysis was performed using a Q Exactive HF-X mass spectrometer equipped with a heated electrospray ionization source. Data were acquired separately in positive and negative ion modes using full-scan/ddMS2 acquisition.

Metabolite detection and annotation were performed using XCMS and the Spectra package by searching public databases, including MoNA, GNPS, HMDB, KEGG, and MS-DIAL LipidBlast, together with an in-house database. Metabolite annotation was further supported by MS1/MS2 spectral matching against multiple metabolite databases, including HMDB, MassBank, Metlin, MoNA, mzVault, KEGG, and the in-house database, and annotation confidence was assigned according to the available spectral matching information. Data quality was evaluated using pooled quality-control samples and internal standards during LC–MS acquisition. Sample reproducibility was assessed by PCA, hierarchical clustering analysis, and Pearson correlation analysis based on metabolite abundance profiles. Differential metabolites between the S and S+R+MT groups were identified using Student’s t-test combined with orthogonal partial least squares discriminant analysis (OPLS-DA). Raw p values were adjusted for multiple testing using the Benjamini–Hochberg false discovery rate (FDR) procedure, and metabolites with VIP > 1 and FDR-adjusted *p* < 0.05 were considered significantly differential. The OPLS-DA model was evaluated by internal cross-validation and 200 permutation tests, and R^2^X, R^2^Y, and Q^2^ were used to assess model performance. KEGG pathway enrichment analysis of differential metabolites was performed using Fisher’s exact test, followed by Benjamini–Hochberg FDR correction, and pathways with FDR-adjusted *p* < 0.05 were considered significantly enriched. For visualization and interpretation, representative metabolites were further selected according to fold change, functional annotation, KEGG enrichment, and biological relevance to salt-stress responses.

**Transcriptome sequencing and DEG analysis.** Leaf samples from the S and S+R+MT groups were also subjected to transcriptome analysis. Total RNA was extracted from frozen leaf tissue, and RNA quality and concentration were evaluated before library construction. mRNA was enriched from total RNA using oligo-dT magnetic beads and then fragmented. First-strand cDNA was synthesized using fragmented mRNA as the template, followed by second-strand cDNA synthesis, end repair, adapter ligation, fragment size selection, and PCR amplification. The constructed libraries were evaluated using an Invitrogen Qubit 3.0 fluorometer and an Agilent 2100 Bioanalyzer. Qualified libraries were sequenced on an Illumina platform using a paired-end 150 bp sequencing strategy.

Raw reads were first evaluated using FastQC. Adapter sequences, low-quality terminal sequences with Q < 15, and reads shorter than 40 bp after trimming were removed using TrimGalore to obtain clean reads. Sequencing quality was evaluated based on raw and clean read numbers, Q20/Q30 values, base composition, and sequencing quality distribution. Clean reads were mapped to the *Zea mays* reference genome database (zma) using STAR, and transcriptome library quality was further assessed using RSeQC. Gene expression levels were quantified using StringTie. Differentially expressed genes between the S and S+R+MT groups were identified using DESeq2 with the thresholds of |log_2_FoldChange| > 1 and adjusted *p* < 0.05. GO and KEGG enrichment analyses of DEGs were performed using ClusterProfiler, and significantly enriched terms or pathways were identified based on *p* < 0.05 or adjusted *p* < 0.05. Gene set enrichment analysis was also performed to support pathway-level interpretation. Detailed sequencing statistics, including raw reads, clean reads, Q30 values, and genome alignment rates, are provided in Supplementary Table S1.

**Integrated transcriptome–metabolome analysis.** Integrated transcriptome–metabolome analysis was performed to identify coordinated molecular changes associated with the improved salt tolerance of maize seedlings under the combined PGPR and MT treatment. The analysis focused on the comparison between the S and S+R+MT groups, which was consistent with the metabolomic and transcriptomic experimental design. Differentially expressed genes and differential metabolites were first mapped to KEGG pathways. Shared pathways enriched in both datasets were identified by comparing the KEGG enrichment results of DEGs and differential metabolites. Pathways related to salt-stress responses, including amino acid metabolism, carbohydrate metabolism, glutathione and ascorbate-related redox metabolism, phenylpropanoid biosynthesis, and MAPK signaling, were further selected for biological interpretation.

Differentially expressed genes and differential metabolites were mapped to KEGG pathways. Shared pathways enriched in both datasets were identified and summarized into functional modules based on pathway annotation and biological relevance. These modules included osmotic adjustment and amino acid-related metabolism, carbohydrate metabolism and carbon allocation, redox buffering and antioxidant metabolism, and stress signaling and defense-related secondary metabolism. The direction of change in genes and metabolites was determined according to log_2_FoldChange values between the S+R+MT and S groups. The integrated framework was used as a pathway-level and module-level interpretive approach to support the physiological findings, rather than as evidence for direct regulatory interactions.

### Statistical Analysis

All data were presented as means ± standard deviation (SD) from three biological replicates. Statistical analyses were performed using IBM SPSS Statistics (version 27.0). For the preliminary experiment, one-way analysis of variance (ANOVA) followed by Tukey’s honestly significant difference (HSD) test was used to determine significant differences among melatonin treatments.

For the factorial experiment, a three-way ANOVA was conducted to evaluate the individual effects of salt stress, PGPR inoculation, and melatonin application, as well as their two-way and three-way interactions. Before ANOVA, data were examined for normality using the Shapiro–Wilk test and for homogeneity of variance using Levene’s test. When significant effects were detected, Tukey’s HSD test was performed for multiple comparisons among treatment means. Statistical significance was defined at *p* < 0.05. Three-way ANOVA results have been provided in Supplementary Table S2.

## Results

### Selection of the Optimal MT Concentration for Salt-Stress Alleviation

Under salt stress, compared with the treatment without MT application, all tested MT concentrations increased maize seedling plant height and chlorophyll content to varying degrees (Fig. S1–S2). Specifically, 50, 100, 150, and 200 μM MT increased chlorophyll content by 12.7%, 23.8%, 14.5%, and 4.27%, respectively (Fig. S2), while plant height increased by 16.9%, 63.4%, 49.1%, and 30.6%, respectively (Fig. S1). Among the tested concentrations, 100 μM MT showed the strongest effect and was therefore selected for subsequent experiments.

### PGPR-Based Treatment Increased Root-Associated Culturable Bacterial Populations under Salt Stress

Different lowercase letters indicate significant differences among treatments according to one-way ANOVA followed by Tukey’s HSD test (*p* < 0.05). Root-associated culturable bacterial populations were enumerated at the end of the 14-d treatment period. In the non-inoculated S group, culturable bacteria were detected at a low background level of approximately 10^2^ CFU g^−1^ fresh root. In contrast, both PGPR-inoculated treatments showed substantially higher culturable counts, reaching approximately 10^4^ CFU g^−1^ fresh root (Table 2). The S+R and S+R+MT groups exhibited comparable culturable bacterial levels, indicating that foliar MT application did not markedly alter root-associated culturable bacterial abundance. These results indicate higher total culturable root-associated bacterial populations in the PGPR-inoculated groups, while S+R and S+R+MT did not differ significantly from each other.

### Combined PGPR and MT Treatment Promotes Growth Recovery in Salt-Stressed Maize Seedlings

Because the experiment followed a 2 × 2 × 2 factorial design, three-way ANOVA was performed to determine the main and interactive effects of salt stress, PGPR inoculation, and melatonin application on physiological and biochemical traits. The detailed ANOVA results are presented in Table S2. Salt stress exerted the major factor contributing to variation on most measured traits, whereas PGPR and MT significantly modulated maize responses under NaCl-induced salinity stress.

Salt treatment significantly inhibited maize seedling growth and root activity (Fig. 1; Fig. S3). Compared with the CK group, seedlings subjected to salt stress (S) exhibited obvious dwarfing, reduced biomass accumulation, and decreased root activity. Compared with the S group, the combined treatment (S+R+MT) significantly increased plant height, root length, fresh weight, dry weight, and root activity by 72.8%, 31.8%, 57.3%, 67.9%, and 68.3%, respectively (Fig. 1A–D; Fig. S3). Tukey’s HSD analysis further showed that S+R+MT resulted in significantly higher plant height and dry weight than both S+R and S+MT treatments, whereas responses of root length, fresh weight, and root activity were comparable to individual PGPR or MT treatments.

### Combined PGPR and MT Treatment Preserves Photosynthetic Pigment Accumulation under Salt Stress

Salt stress significantly reduced photosynthetic pigment accumulation, with chlorophyll a, chlorophyll b, and carotenoid contents decreasing by 29.1%, 35.4%, and 32.7%, respectively, compared with the CK group (Fig. 2A–2H). Under non-stress conditions, CK+R+MT treatment moderately enhanced pigment accumulation, increasing chlorophyll a, chlorophyll b, and carotenoid contents by 12.7%, 20.1%, and 20.3%, respectively. Under salt stress, S+R+MT treatment showed the strongest recovery effect, increasing chlorophyll a, chlorophyll b, and carotenoid contents by 27.1%, 70.5%, and 80.3%, respectively, compared with the S group. Three-way ANOVA revealed a significant Salt × PGPR × MT interaction for chlorophyll a (Table S2), indicating that the combined application of PGPR and MT was particularly involved in maintaining chlorophyll a stability under saline conditions. Tukey’s HSD analysis further confirmed that S+R+MT significantly enhanced chlorophyll a accumulation compared with individual PGPR or MT treatments. Consistently, total chlorophyll content was 45.7% higher in the S+R+MT group than in the S group (Fig. 2I). Together, these results indicate that PGPR and MT jointly contributed to preserving photosynthetic pigment accumulation and alleviating salt-induced damage to the photosynthetic apparatus.

### Combined PGPR and MT Treatment Strengthens Osmotic Adjustment under Salt Stress

Fig. 3A–B show the changes in proline and soluble sugar contents under different treatments. Under non-saline conditions, proline and soluble sugar contents remained relatively low in maize seedlings. Salt treatment increased both osmolytes, with the S group showing 8.46% and 27.9% higher proline and soluble sugar contents, respectively, than the CK group. Under salinity, proline content further increased by 19.1%, 10.1%, and 28.7% in the S+R, S+MT, and S+R+MT groups, respectively, relative to the S group (Fig. 3A). Proline accumulation showed a significant Salt × PGPR interaction (Table S2). However, Tukey’s HSD test showed that the proline level in S+R+MT was not significantly different from that in S+R, suggesting that PGPR played an important role in regulating proline accumulation under saline conditions.

Likewise, soluble sugar content increased by 28.9%, 44.2%, and 61.6% in the S+R, S+MT, and S+R+MT treatments, respectively (Fig. 3B). The S+R+MT treatment exhibited significantly higher soluble sugar accumulation than S, S+R, and S+MT treatments, indicating that combined PGPR and MT application provided a stronger effect on soluble sugar-mediated osmotic adjustment. Together, these results suggest that PGPR and MT regulated osmotic adaptation through partially distinct mechanisms, with their combined application showing stronger effects particularly on soluble sugar accumulation.

### Combined PGPR and MT Treatment Restricts Salt-Induced Oxidative Damage

Salt stress caused marked increases in MDA and H_2_O_2_ contents (Fig. 4A and 4B). Compared with the CK group, the S group exhibited 76.3% and 91.1% increases in MDA and H_2_O_2_ contents, respectively, indicating severe oxidative damage under saline conditions. Significant Salt × PGPR and Salt × MT interactions were observed for MDA and H_2_O_2_ accumulation (Table S2), suggesting that PGPR and MT were involved in regulating oxidative stress responses under salinity.

Under non-stress conditions, CK+R+MT treatment reduced MDA and H_2_O_2_ contents by 28.7% and 17.6%, respectively, compared with CK. Under salt stress, the S+R+MT treatment significantly decreased MDA and H_2_O_2_ contents by 27.4% and 37.9%, respectively, relative to the S group. Furthermore, Tukey’s HSD analysis demonstrated that S+R+MT exhibited significantly lower MDA and H_2_O_2_ levels than both S+R and S+MT treatments, indicating that the combined application of PGPR and MT provided enhanced protection against salt-induced oxidative damage. These results suggest that PGPR and MT jointly contributed to maintaining reactive oxygen species homeostasis and protecting membrane integrity under saline conditions.

### Combined PGPR and MT Treatment Reinforces Antioxidant Defense under Salt Stress

Under non-saline conditions, POD, SOD, CAT, and APX activities in maize seedlings remained relatively low and stable (Fig. 4C–4F). The CK+R+MT treatment increased POD, SOD, CAT, and APX activities by 18.1%, 15.1%, 40.6%, and 12.2%, respectively, suggesting that combined PGPR and MT application moderately enhanced basal antioxidant capacity even under non-stress conditions.

Under salt stress, antioxidant enzyme responses showed differential patterns. SOD activity showed a slight increase in the S group, whereas CAT and APX activities decreased significantly, indicating an imbalance in antioxidant regulation under salinity (Fig. 4C–4F). Notably, CAT activity showed a significant Salt × PGPR × MT interaction (Table S2), suggesting that combined PGPR and MT regulation was particularly involved in restoring CAT-mediated antioxidant defense.

Compared with the S group, PGPR or MT application partially restored antioxidant enzyme activities. PGPR treatment increased POD, CAT, and APX activities by 13.3%, 44.6%, and 37.5%, respectively, whereas MT application increased these activities by 30.8%, 64.9%, and 20.3%, respectively. The S+R+MT treatment exhibited the greatest increases compared with S, increasing POD, SOD, CAT, and APX activities by 42.3%, 14.3%, 95.8%, and 61.7%, respectively, compared with S. Tukey’s HSD analysis further confirmed that S+R+MT significantly increased CAT and APX activities compared with both S+R and S+MT treatments, indicating superior activation of antioxidant defense by the combined treatment. Together with the reduced accumulation of MDA and H_2_O_2_, these findings demonstrate that PGPR and MT enhanced salt tolerance by improving antioxidant buffering capacity and maintaining reactive oxygen species homeostasis.

### Combined PGPR and MT Treatment Induces Metabolic Reprogramming under Salt Stress

Untargeted metabolomic profiling detected 836 differential metabolites between the S+R+MT and S groups after FDR correction, of which 456 were upregulated and 380 were downregulated in the S+R+MT group relative to the S group (Fig. 5A). The volcano plot revealed a clear treatment-associated metabolic separation. In this analysis, the size of each point reflected the corresponding VIP value, indicating the contribution of individual metabolites to group discrimination. As shown in Figure 5B, KEGG enrichment analysis indicated that these differential metabolites were mainly enriched in biosynthesis of amino acids, the pentose phosphate pathway, glycine, serine and threonine metabolism, tyrosine metabolism, nicotinate and nicotinamide metabolism, nucleotide metabolism, purine metabolism, and ABC transporters.

To further illustrate the metabolic differences between the S and S+R+MT groups, 28 representative differential metabolites were selected for hierarchical clustering analysis. As shown in Fig. 6, these metabolites were mainly associated with amino acid metabolism, sugar metabolism, phenolic acid metabolism, and antioxidant-related processes. Among them, L-proline, glucose, sucrose, dehydroascorbic acid, 4-hydroxycinnamic acid, and ferulic acid differed markedly between the two groups. PCA based on all differential metabolites also showed an overall separation trend between the S and S+R+MT groups (Fig. S4).

The OPLS-DA model yielded R^2^X(cum) = 0.679, R^2^Y(cum) = 1.000, and Q^2^(cum) = 0.987. The 200-permutation analysis yielded pR^2^Y = 0.145 and pQ^2^ = 0.145; given the limited number of biological replicates, these results were interpreted cautiously. Overall, the metabolomic data point to broad shifts in osmotic adjustment, carbon metabolism, redox-related pathways, and defense-associated metabolites in response to the combined treatment.

### Combined PGPR and MT Treatment Is associated with Transcriptomic Changes under Salt Stress

Transcriptome profiling revealed marked gene-expression differences between the S+R+MT and S groups, with 7307 differentially expressed genes identified in total, including 3778 upregulated and 3529 downregulated genes in the S+R+MT group (Fig. 7A). The volcano plot showed a clear shift in transcriptional patterns under the combined treatment. KEGG enrichment analysis further indicated that these DEGs were mainly associated with carbon metabolism, amino acid biosynthesis, plant–pathogen interaction, MAPK signaling, phenylpropanoid biosynthesis, starch and sucrose metabolism, glycolysis/gluconeogenesis, cysteine and methionine metabolism, and alanine, aspartate, and glutamate metabolism (Fig. 7B). These results indicate that enhanced salt tolerance under the combined treatment was accompanied by extensive transcriptional changes involving multiple stress-responsive pathways. Because transcriptomic analysis was conducted only between S and S+R+MT treatments, these changes represent transcriptional responses associated with the combined treatment rather than evidence distinguishing PGPR- or MT-specific effects. GO enrichment analysis further supported the involvement of stress-responsive biological processes, cellular components, and molecular functions in the transcriptional response to the combined treatment (Fig. S5).

3.10. Integrated Transcriptome–Metabolome Analysis Reveals Pathway-Level Responses associated with the Combined Treatment

To further characterize molecular changes associated with the combined treatment, an integrated transcriptome–metabolome framework was constructed based on shared pathway enrichment patterns between differentially expressed genes and metabolites (Fig. 8). Four major modules were identified, including osmotic adjustment/amino acid-related metabolism, carbohydrate metabolism/carbon allocation, redox buffering/glutathione–AsA-related metabolism, and stress signaling/phenylpropanoid-related defense. The osmotic adjustment/amino acid-related module showed coordinated changes in amino acid-associated pathways at both transcriptomic and metabolomic levels, accompanied by increased accumulation of L-proline and L-cysteine. The carbohydrate metabolism/carbon allocation module was characterized by enrichment of carbohydrate-related pathways and increased levels of glucose and sucrose. The redox buffering/glutathione–AsA-related module exhibited coordinated changes in redox-associated pathways, together with alterations in dehydroascorbic acid and L-cysteine levels. The stress signaling/phenylpropanoid-related defense module was associated with changes in phenylpropanoid-related pathways and increased accumulation of 4-hydroxycinnamic acid and ferulic acid.

Overall, the integrated analysis suggests that the improved performance under combined treatment was associated with coordinated transcriptomic and metabolic adjustments across multiple functional pathways. This integrative analysis provides a pathway-level overview of molecular changes associated with the combined treatment, rather than demonstrating direct regulatory relationships or combination-specific mechanisms.

## Discussion

### Combined PGPR and MT Promote Growth Recovery under Salinity by Integrating Complementary Protective Functions

Salinity severely constrains plant growth and productivity through osmotic stress and oxidative injury [29]. In the present study, 100 mM NaCl markedly inhibited plant height, root length, biomass accumulation, and root activity in maize seedlings, confirming that maize is highly sensitive to salinity at the seedling stage. In contrast, the combined PGPR and MT treatment significantly improved these growth-related parameters, indicating that the combined treatment exerted a stronger alleviating effect on salt stress-induced growth inhibition than either individual treatment. The improvement in root activity suggests that the combined treatment may help maintain root physiological function under salt stress, which may contribute to improved whole-seedling stress adaptation [30].

For a PGPR-based biostimulant, efficacy depends not only on intrinsic plant growth-promoting traits but also on persistence in the root-associated environment under stress. The enumeration of root-associated culturable bacterial populations was therefore included to support the microbiological component of the treatment system. MT has been reported to alleviate salt stress by enhancing antioxidant capacity and reducing oxidative damage [31], whereas PGPR mainly exerts a more sustained protective effect by improving the rhizosphere microenvironment, enhancing nutrient utilization, and promoting root growth [32]. The better growth recovery under co-application likely reflects the complementary functions of PGPR and MT. PGPR are generally associated with improved root function, nutrient acquisition, and rhizosphere-mediated stress mitigation [33], whereas melatonin is more closely linked to rapid antioxidant protection and redox regulation [13]. Their combined use may therefore reduce cellular damage while also supporting growth-related processes, which is consistent with the higher plant height, biomass, and root activity observed in this study.

### Combined PGPR and MT Help Preserve Photosynthetic Pigment Stability under Salt Stress

Salinity severely constrains plant growth, largely because salinity can impair chloroplast structure and photosynthetic machinery through osmotic and oxidative stress, thereby promoting pigment loss [34]. Chlorophyll a is the major chlorophyll directly involved in reaction-center photochemistry, whereas chlorophyll b mainly participates in peripheral light harvesting and excitation energy transfer [35, 36]. Carotenoids function not only as accessory pigments for light capture but also as key photoprotective components by dissipating excess excitation energy and quenching ROS[37]. Thus, preservation of photosynthetic pigments is an important component of plant salt tolerance.

Exposure to 100 mM NaCl led to pronounced declines in chlorophyll a, chlorophyll b, and carotenoid contents in maize seedlings, suggesting substantial impairment of the photosynthetic apparatus. Previous work has linked this reduction in chlorophyll to disturbed chlorophyll metabolism, particularly inhibited pigment biosynthesis and lower activity of key enzymes in the chlorophyll biosynthetic pathway[38]. In addition, salt stress-triggered phytohormonal reprogramming may further affect photosynthesis and pigment metabolism[39]. Exogenous MT has been reported to improve photosynthetic performance and maintain pigment stability under salt stress by regulating photosynthesis-related genes and stabilizing the photosynthetic system[40]. Likewise, beneficial rhizobacteria such as Pseudomonas can promote plant growth and increase photosynthetic pigment contents under saline conditions [41]. In this study, the S+R+MT treatment exhibited the highest pigment contents under salt stress, suggesting that the combined PGPR and MT treatment may have provided stronger protection to the photosynthetic system than either treatment alone. This result is consistent with previous reports showing that the combined ACC deaminase-producing rhizobia and MT treatment increased chlorophyll content in common bean [42], and that rhizobial inoculation together with exogenous MT mitigated the decline in chlorophyll and carotenoid contents in Medicago truncatula under abiotic stress [19].

The higher chlorophyll and carotenoid contents under the combined treatment may be related, at least in part, to better redox control under salinity. Excess ROS can damage chloroplast membranes and pigment–protein complexes, accelerating pigment loss and reducing light-harvesting efficiency. Therefore, the pigment stability observed here likely reflects improved protection of the photosynthetic apparatus under combined PGPR and MT treatment.

### Combined PGPR and MT Strengthen Osmotic Adjustment in Salt-Stressed Maize Seedlings

Proline and soluble sugars play key roles in osmotic adjustment under salt stress by helping maintain cellular water balance, stabilizing membranes, and reducing oxidative injury [43]. Under saline conditions, plants often cope with osmotic injury by accumulating osmolytes such as proline and soluble sugars. In the present study, compared with the CK group, salt stress significantly increased the contents of proline and soluble sugars in maize seedlings, indicating the activation of osmotic adjustment in response to salt-induced stress. A similar trend was reported by Liu *et al*. [44], who observed greater accumulation of osmotic adjustment substances in alfalfa under alkaline stress. In our study, the combined treatment further increased proline and soluble sugar contents by 28.7% and 61.6%, respectively, relative to the S group. A similar pattern has been observed in earlier studies: Khan *et al*. [45] showed that Pseudomonas fluorescens and MT jointly increased proline accumulation in mustard, while Irshad *et al*. [19] demonstrated that in Medicago truncatula under abiotic stress, rhizobial inoculation combined with MT promoted the accumulation of osmolytes.

Proline and soluble sugars are not only compatible solutes but also contributors to membrane stabilization and metabolic buffering under stress [46]. In the present study, their further accumulation in the S+R+MT group suggests a stronger osmotic adjustment response than that observed under salt stress alone. This enhanced osmolyte accumulation may have helped reduce osmotic injury and support membrane stability in maize seedlings.

### Combined PGPR and MT Reinforce Redox Buffering by Enhancing Antioxidant Defense

Under NaCl stress, maize seedlings accumulate excessive ROS, and overproduction of Q_2_^.–^ and H_2_O_2_ is a major cause of oxidative damage [4]. In the present study, 100 mM NaCl significantly increased the contents of MDA and H_2_O_2_ in maize seedlings, indicating that salt stress induced pronounced oxidative stress. This finding agrees with earlier studies showing that excessive NaCl markedly aggravates membrane lipid peroxidation in wheat, disrupts ROS homeostasis, and further impairs the stability of cellular membrane systems [47]. Exogenous application of MT has been found to reduce ROS buildup, stimulate antioxidant enzyme activity, and thereby strengthen the oxidative defense capacity of plants under salt stress[48]. Consistent with this, exogenous MT has also been reported to alleviate oxidative injury in apple rootstock seedlings under saline-alkali stress by strengthening antioxidant defense and maintaining physiological homeostasis [49]. Studies in tomato have shown that plant growth-promoting rhizobacteria can enhance oxidative defense and thereby improve salt tolerance through changes in the rhizosphere environment and hormone signaling [50]. In this study, the S+R+MT treatment exhibited the most pronounced alleviating effect, as reflected by the greatest reductions in MDA and H_2_O_2_ contents. Taken together, these observations suggest that the combined PGPR and MT treatment offered stronger protection against salt-induced oxidative injury than either treatment applied alone.

In plants, the antioxidant enzyme system constitutes a crucial defense network for scavenging ROS and maintaining cellular redox homeostasis, with SOD, CAT, APX, and POD representing its core components. SOD catalyzes the dismutation of Q_2_^.–^ to H_2_O_2_ and O_2_, whereas POD, CAT, and APX subsequently participate in H_2_O_2_ scavenging and detoxification [51]. Among them, APX, a key enzyme in the AsA–GSH cycle, mainly catalyzes the ascorbate-dependent reduction of H_2_O_2_ and plays an important role in maintaining redox balance and alleviating lipid peroxidation damage [52]. Under salinity, several key antioxidant enzymes showed varying degrees of increase after individual PGPR or MT treatment, whereas the combined PGPR+MT treatment produced the strongest enhancement, consistent with the observed decreases in MDA and H_2_O_2_ contents. Earlier work has shown that melatonin markedly increases SOD, POD, CAT, and APX activities in cucumber seedlings [40].

A notable feature of the combined treatment was that it enhanced several antioxidant enzymes simultaneously rather than affecting only one part of the antioxidant defense network [51]. Because SOD, CAT, POD, and APX function cooperatively in ROS detoxification [40], their concurrent increase is consistent with more effective redox regulation under salt stress. This may explain why the S+R+MT treatment was associated with lower MDA and H_2_O_2_ contents than the individual treatments.

### Integrated Transcriptome–Metabolome Analysis Identifies Stress-Responsive Pathways associated with the Combined Treatment

Transcriptomic and metabolomic data provided additional support for the physiological responses observed under the combined treatment. The superior performance of S+R+MT was associated with broad changes in stress-related metabolic and transcriptional processes. These changes were mainly reflected in osmotic adjustment, carbon metabolism, redox buffering, and defense-related secondary metabolism. At the metabolite level, the S+R+MT treatment resulted in 836 differential metabolites relative to the S group after FDR correction, indicating that the combined treatment markedly altered the metabolic composition of maize leaves and the overall pattern of metabolic responses. Among these, 28 representative differential metabolites were further selected, mainly involving amino acid metabolism, sugar metabolism, phenolic acid metabolism, and antioxidant-related processes, suggesting that the stronger protective effect of the combined treatment was associated with adjustments in osmotic regulation, redox homeostasis, and secondary metabolism [53, 54]. In particular, significant changes in L-proline, glucose, and sucrose were consistent with a marked remodeling of osmotic and carbohydrate metabolism under the combined treatment [55]. Meanwhile, the change in dehydroascorbic acid was consistent with the involvement of antioxidant-related metabolism and the AsA–GSH redox system, which may have contributed to the enhanced ROS-scavenging capacity and the reduced MDA and H_2_O_2_ levels observed under the combined treatment [52, 56]. Taken together, these transcriptomic and metabolomic changes provide molecular support for the improved salt tolerance observed under the combined PGPR and MT treatment. However, because the omics analyses were conducted only between the S and S+R+MT groups, these results should be interpreted as a molecular characterization of the combined treatment response relative to salt stress alone, rather than direct evidence of molecular synergism between PGPR and MT. Future omics analyses including S+R and S+MT treatments will be required to distinguish treatment-specific responses and interactive molecular effects.

However, because transcriptomic and metabolomic analyses were performed only between the S and S+R+MT treatments, these datasets cannot distinguish the individual contributions of PGPR and melatonin or determine whether specific pathways are uniquely induced by their interaction. It should also be noted that MT and PGPR were applied at different spatial compartments in this study. While MT was directly applied to leaves, PGPR primarily acted in the root-associated environment, and transcriptomic and metabolomic analyses were performed using leaf tissues. Therefore, the omics results should be interpreted as leaf molecular responses associated with the combined treatment rather than direct evidence distinguishing the individual contributions of foliar MT and root-associated PGPR.

These metabolic shifts were generally in line with the physiological responses observed in this study. The enrichment of amino acid biosynthesis is consistent with broad changes in amino acid-related stress metabolism. Meanwhile, enrichment of the pentose phosphate pathway suggests adjustments in carbon metabolism and reducing-power supply under salinity [52]. In particular, the pentose phosphate pathway is a major source of NADPH, which is indispensable for antioxidant recycling and redox buffering [57]. Likewise, changes in nicotinate and nicotinamide metabolism may reflect adjustments in NAD(P)(H)-dependent processes, further supporting the maintenance of intracellular redox balance [58].

These metabolite-level changes should be considered together with the transcriptomic results, which showed related shifts across several of the same functional processes. Transcriptome analysis revealed extensive changes in gene expression under the combined treatment, and the identified DEGs were mainly associated with pathways related to carbon metabolism, amino acid biosynthesis, MAPK signaling, phenylpropanoid biosynthesis, starch and sucrose turnover, glycolytic/gluconeogenic processes, and cysteine and methionine metabolism. These transcriptional changes agree with the metabolic adjustments observed here and are also supported by recent transcriptome–metabolome studies showing that salt adaptation is associated with coordinated changes in gene expression, carbon/nitrogen metabolism, and stress-associated secondary metabolism [59–61]. In particular, the higher transcript abundance of PFK5 and CWINV3 was broadly consistent with the altered levels of glucose and sucrose, supporting active adjustment of the carbohydrate metabolism / carbon allocation module under salt stress, a pattern also reported in recent multi-omics studies of salinity adaptation [61, 62]. Likewise, the coordinated changes in GRX1, 1-Cys Prx, L-cysteine, and dehydroascorbic acid suggest reinforcement of the redox buffering / glutathione–AsA-related metabolism module, in agreement with previous reports highlighting the importance of glutathione metabolism and antioxidant buffering in integrated salt-stress responses [59, 61, 62]. The integrated transcriptome–metabolome analysis further revealed coordinated changes in several stress-responsive functional modules, including osmotic adjustment/amino acid-related metabolism and phenylpropanoid-related defense. These pathway-level responses were accompanied by changes in related metabolites, such as L-proline, L-cysteine, 4-hydroxycinnamic acid, and ferulic acid, suggesting that combined PGPR and MT treatment was associated with broad metabolic and transcriptional reprogramming under salt stress [61, 63].

Overall, the improved salt tolerance under co-application was associated with linked changes in physiology, metabolism, and gene expression. The main responses involved osmotic adjustment, carbon metabolism, redox buffering, and defense-related secondary metabolism. These physiological and molecular changes support the potential application of PGPR-based microbial biostimulants combined with plant signaling regulators such as melatonin for improving crop performance under saline conditions.

Although the present results demonstrate that the combined PGPR+MT treatment was associated with enhanced salt tolerance, a limitation of this study is that strain-specific root colonization by the inoculated bacteria under salt-stress conditions was not directly quantified. The strains used here were previously isolated from a saline-alkali environment and exhibit halotolerant traits [21], and the hydroponic system included repeated re-inoculation to maintain bacterial density. The CFU assay performed here estimated total culturable root-associated bacterial populations and could not distinguish the inoculated strains from background culturable bacteria. However, future work using strain-specific qPCR, marker-tagged strains, or amplicon-based community profiling will be necessary to link colonization dynamics more directly with the observed physiological benefits.

## Conclusion

In summary, co-application of a defined PGPR inoculant and melatonin alleviated salt-induced damage in maize seedlings more effectively than either treatment alone. This advantage was reflected in improved growth, higher photosynthetic pigment levels, stronger osmotic adjustment, reduced oxidative injury, and enhanced antioxidant enzyme activities. Transcriptomic and metabolomic analyses further indicated that these physiological responses were accompanied by coordinated changes in osmotic regulation, carbon allocation, redox buffering, and defense-related secondary metabolism. Overall, these findings support the potential of PGPR-MT co-application for improving maize seedling performance under hydroponic NaCl stress, while its effectiveness under soil and field conditions remains to be validated. Field application may be constrained by the cost and logistics of repeated PGPR inoculation and MT spraying, particularly under light-avoidance requirements. Moreover, native microbial competition and heterogeneous soil conditions may affect PGPR establishment and treatment efficacy. Future studies should therefore optimize application frequency and formulation and validate the treatment under saline field conditions.

## Supplemental Materials

Supplementary data for this paper are available on-line only at http://jmb.or.kr.



## Figures and Tables

**Fig. 1:**
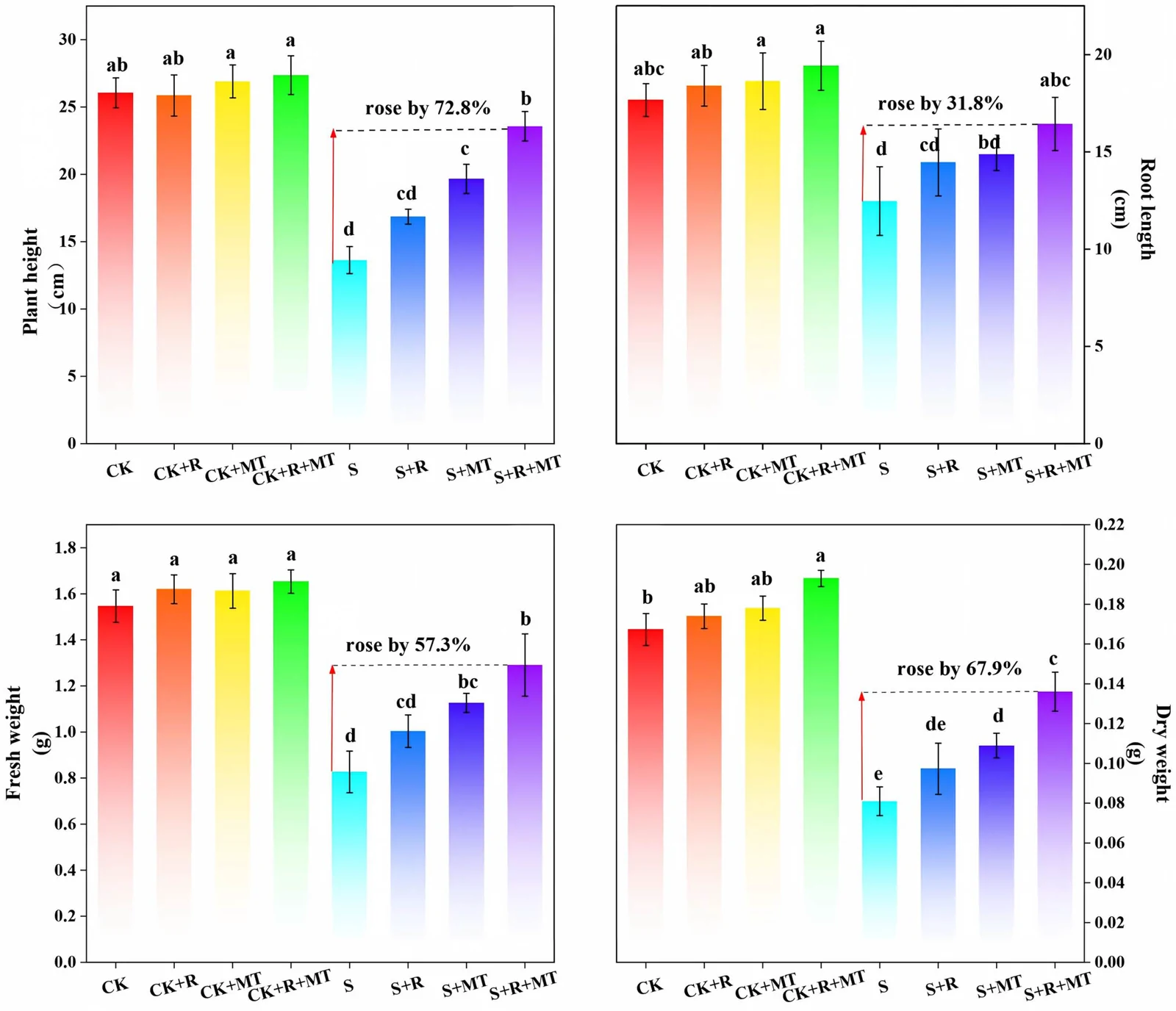
Effects of PGPR inoculation, exogenous melatonin (MT), and their combined treatment on growth performance of maize seedlings under control and salt stress conditions. (**A**) Plant height; (**B**) root length; (**C**) fresh weight; (**D**) dry weight. Data are presented as means ± SD of three biological replicates (n = 3). Different lowercase letters indicate significant differences among treatments according to Tukey’s HSD test (*p* < 0.05). * CK = control; CK+R = control + PGPR inoculation; CK+MT = control + melatonin; CK+R+MT = control + PGPR inoculation + melatonin; S = 100 mM NaCl salt stress; S+R = salt stress + PGPR inoculation; S+MT = salt stress + melatonin; S+R+MT = salt stress + PGPR inoculation + melatonin. Here, R represents inoculation with the mixed PGPR consortium of *Bacillus velezensis* and *Brevibacterium frigoritolerans*, and MT represents melatonin treatment.

**Fig. 2:**
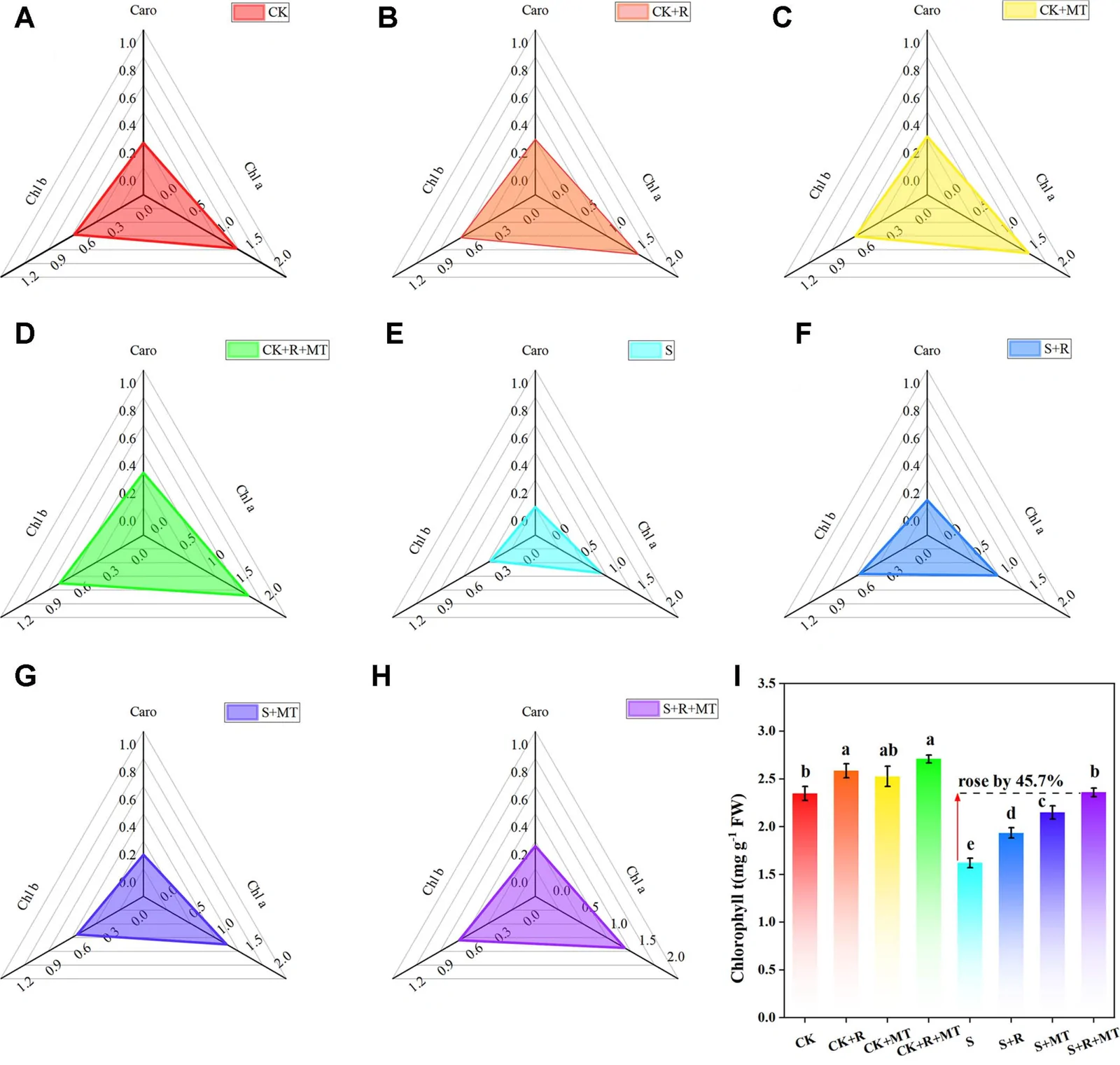
Effects of PGPR inoculation, exogenous melatonin (MT), and their combined treatment on photosynthetic pigment contents in maize seedlings under control and salt stress conditions. (**A–D**) Chlorophyll a, chlorophyll b, and carotenoid contents under control conditions; (**E–H**) chlorophyll a, chlorophyll b, and carotenoid contents under salt stress conditions; (**I**) total chlorophyll content under different treatments. Data are presented as means ± SD of three biological replicates (n = 3). Different lowercase letters indicate significant differences among treatments according to Tukey’s HSD test (*p* < 0.05). For panels **A–H**, the radar-chart axes represent chlorophyll a (Chl a), chlorophyll b (Chl b), and carotenoids (Car), while the individual panels correspond to the respective treatment groups. For panel I, the X-axis treatment labels are CK, CK+R, CK+MT, CK+R+MT, S, S+R, S+MT, and S+R+MT, as defined above.

**Fig. 3:**
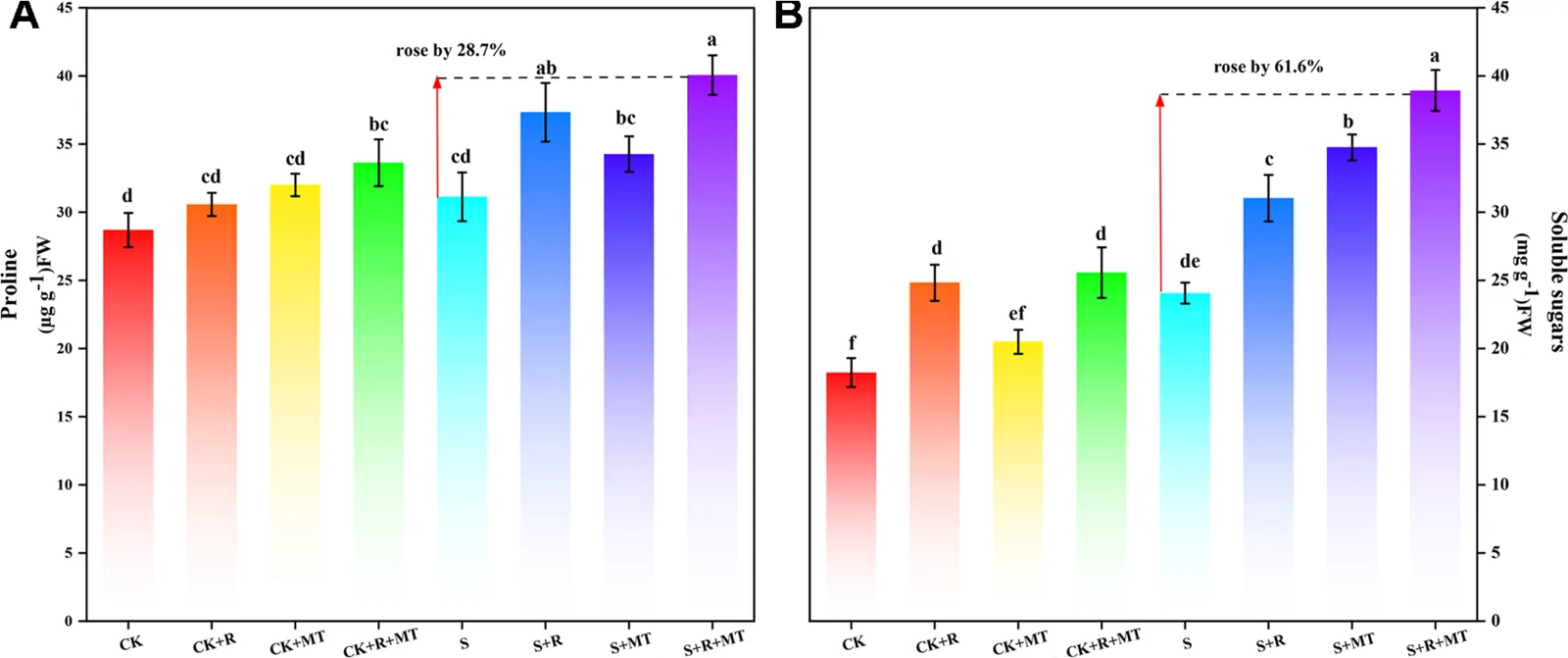
Effects of PGPR inoculation, exogenous melatonin (MT), and their combined treatment on osmotic adjustment substances in maize seedlings under control and salt stress conditions. (**A**) Proline content; (**B**) soluble sugar content. Data are presented as means ± SD of three biological replicates (n = 3). Different lowercase letters indicate significant differences among treatments according to Tukey’s HSD test (*p* < 0.05). * CK = control; CK+R = control + PGPR inoculation; CK+MT = control + melatonin; CK+R+MT = control + PGPR inoculation + melatonin; S = 100 mM NaCl salt stress; S+R = salt stress + PGPR inoculation; S+MT = salt stress + melatonin; S+R+MT = salt stress + PGPR inoculation + melatonin. Here, R represents inoculation with the mixed PGPR consortium of *Bacillus velezensis* and *Brevibacterium frigoritolerans*, and MT represents melatonin treatment.

**Fig. 4:**
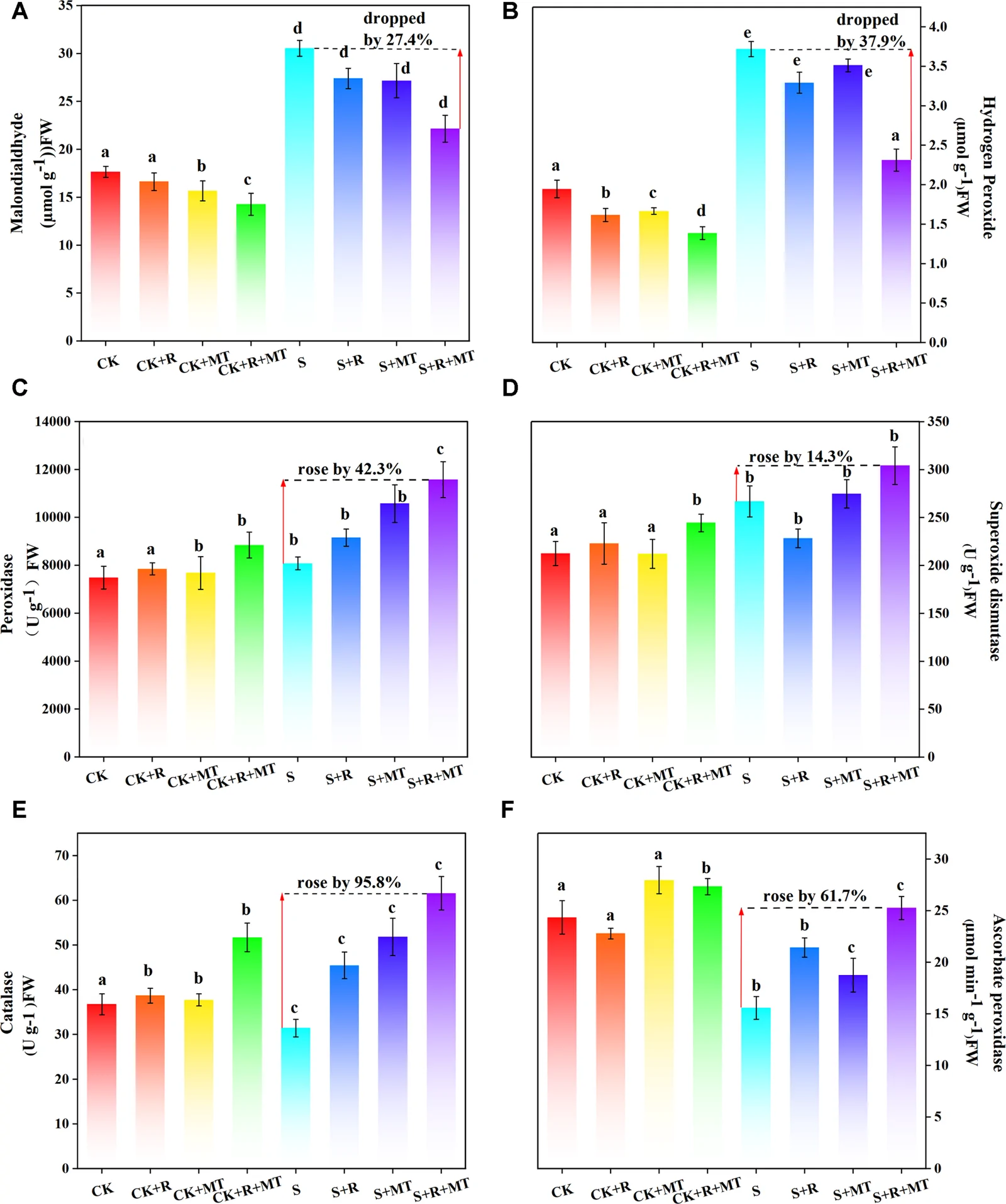
Effects of PGPR inoculation, exogenous melatonin (MT), and their combined treatment on oxidative damage and antioxidant enzyme activities in maize seedlings under control and salt stress conditions. (**A**) Malondialdehyde (MDA) content; (**B**) hydrogen peroxide (H_2_O_2_) content; (**C**) peroxidase (POD) activity; (**D**) superoxide dismutase (SOD) activity; (**E**) catalase (CAT) activity; and (**F**) ascorbate peroxidase (APX) activity. Data are presented as means ± SD of three biological replicates (n = 3). Different lowercase letters indicate significant differences among treatments according to Tukey’s HSD test (*p* < 0.05). * CK = control; CK+R = control + PGPR inoculation; CK+MT = control + melatonin; CK+R+MT = control + PGPR inoculation + melatonin; S = 100 mM NaCl salt stress; S+R = salt stress + PGPR inoculation; S+MT = salt stress + melatonin; S+R+MT = salt stress + PGPR inoculation + melatonin. Here, R represents inoculation with the mixed PGPR consortium of *Bacillus velezensis* and *Brevibacterium frigoritolerans*, and MT represents melatonin treatment.

**Fig. 5:**
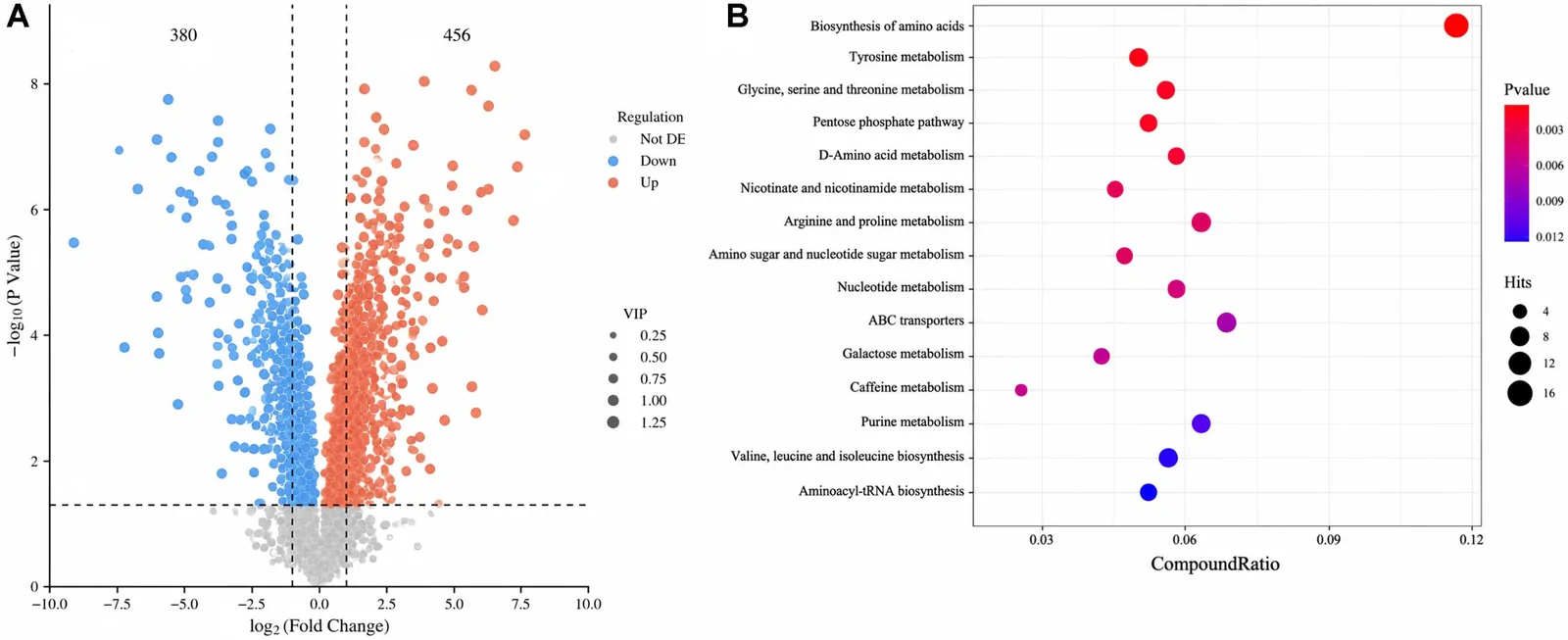
Untargeted metabolomic comparison between the S+R+MT and S treatments. (**A**) Volcano plot of differential metabolites. Red dots indicate significantly upregulated metabolites, blue dots indicate significantly downregulated metabolites, and gray dots indicate non-significant metabolites. Point size reflects the corresponding VIP value. (**B**) KEGG pathway enrichment analysis of the identified differential metabolites. Bubble size represents the number of metabolites enriched in each pathway, and bubble color indicates the enrichment significance level. (**A**) X-axis: log_2_(Fold Change), representing S+R+MT relative to S. (**B**) X-axis: GeneRatio.

**Fig. 6:**
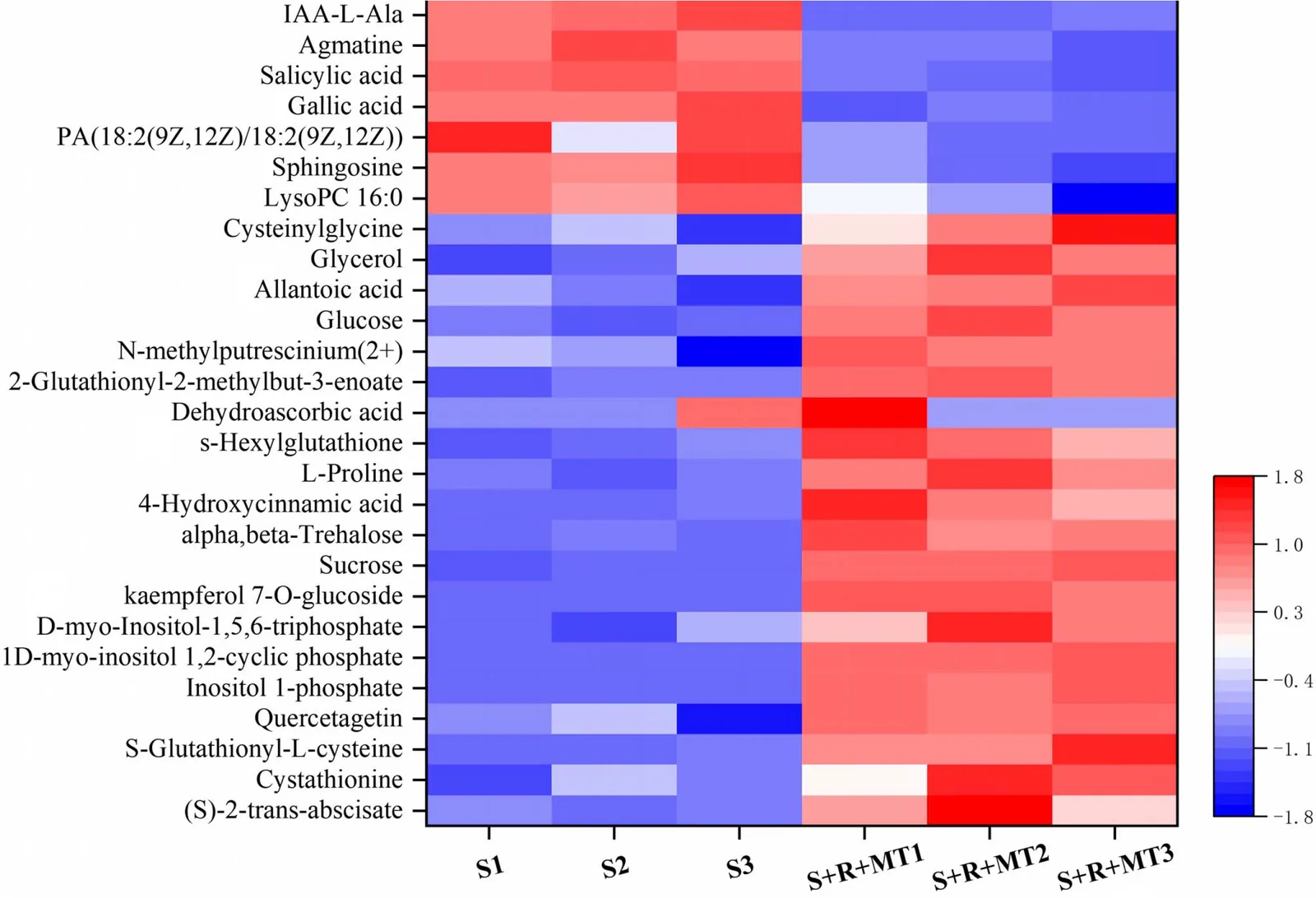
Hierarchical clustering heatmap of 28 representative differential metabolites identified between the S+R+MT and S treatments. Rows represent metabolites and columns represent biological replicates. Red indicates higher relative abundance, whereas blue indicates lower relative abundance. The X-axis represents individual biological replicates: S1–S3 = three biological replicates of the salt-stress treatment; S+R+MT1–S+R+MT3 = three biological replicates of the combined PGPR and melatonin treatment under salt stress.

**Fig. 7:**
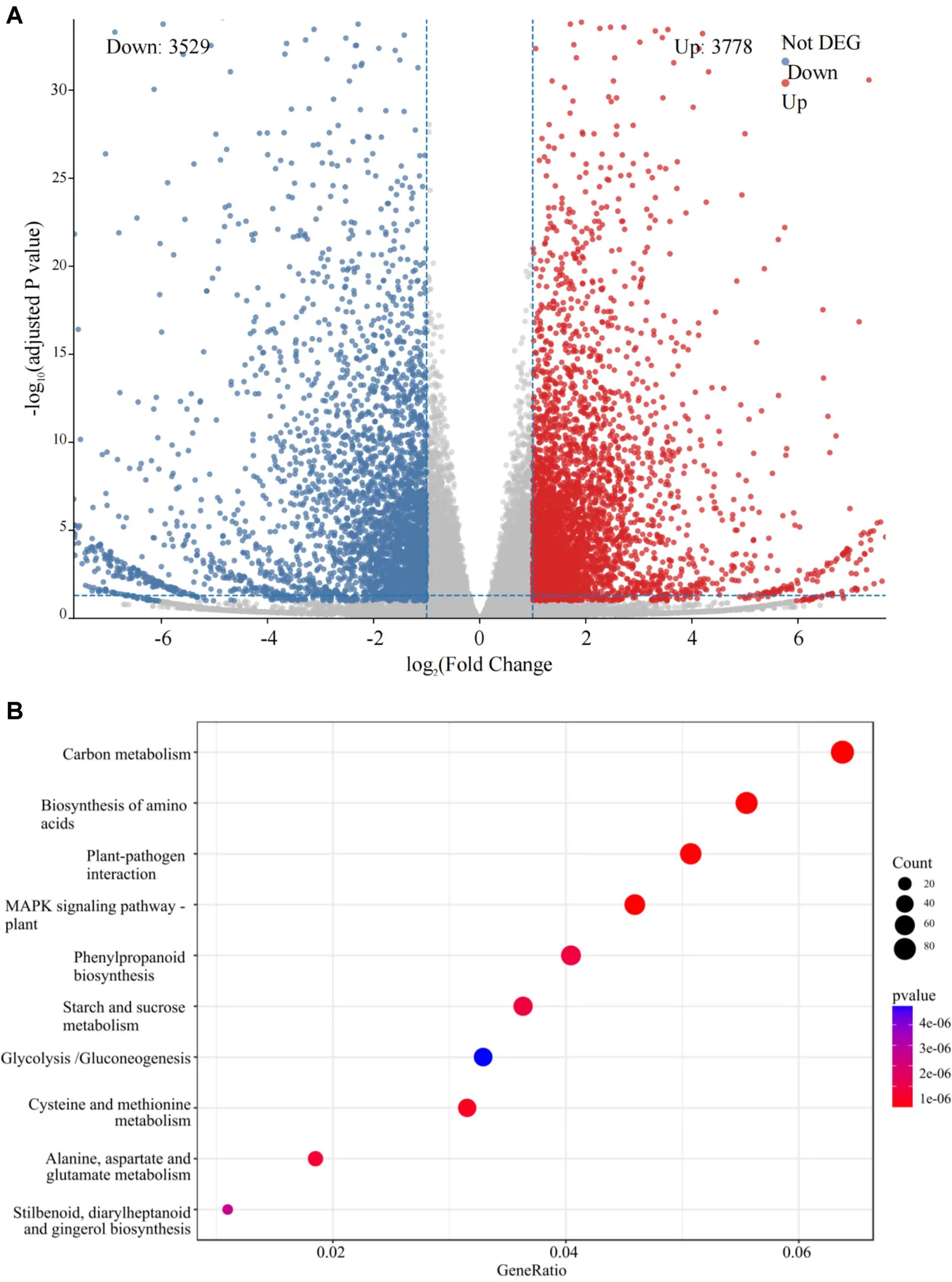
Transcriptomic comparison between the S+R+MT and S treatments. (**A**) Volcano plot of differentially expressed genes (DEGs). Red dots indicate upregulated genes in the S+R+MT group relative to the S group, blue dots indicate downregulated genes, and gray dots indicate non-significant genes. (**B**) KEGG enrichment analysis of the identified DEGs. Bubble size represents the number of genes enriched in each pathway, and bubble color indicates the enrichment significance level.

**Fig. 8:**
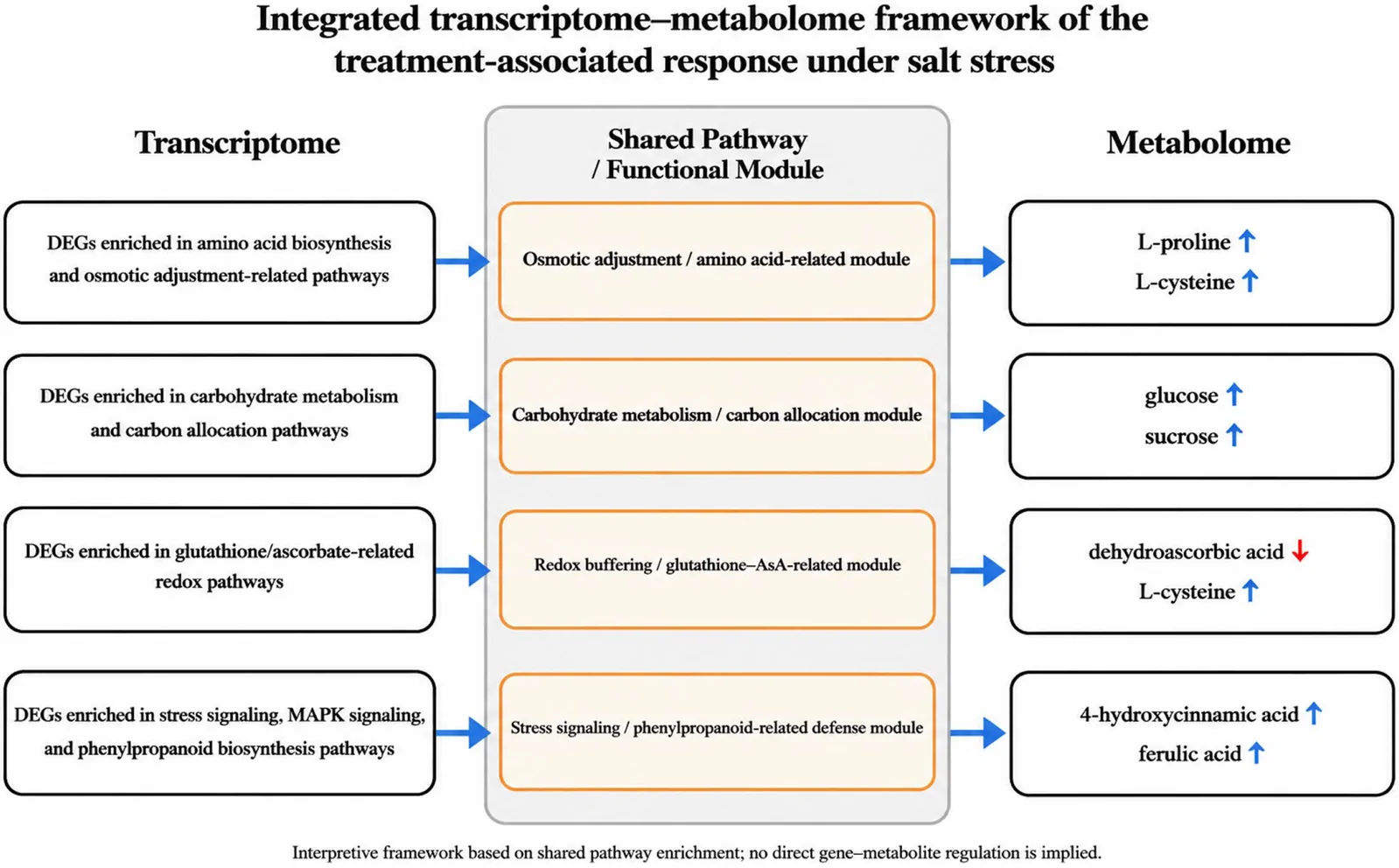
Integrated transcriptome–metabolome framework showing shared pathway responses associated with combined PGPR and melatonin treatment under salt stress. Transcriptomic and metabolomic changes were integrated based on KEGG pathway enrichment and summarized into functional modules related to osmotic adjustment, carbohydrate allocation, redox buffering, and stress-related defense. Arrows indicate metabolite changes in the S+R+MT group relative to the S group. The framework represents pathway-level association rather than direct gene–metabolite regulation.

**Table 1:** Summary of key plant growth-promoting traits of *Bacillus velezensis* and *Brevibacterium frigoritolerans* used in this study.

Trait	*B. velezensis*	*B. frigoritolerans*	Relevance to Salt Stress
ACC Deaminase	+	+	Reduces stress ethylene
IAA Production	+	+	Stimulates root growth
P Solubilization	+	++	Increases P availability
N Fixation	-	±	Contributes to N nutrition

**Table 2:** Total culturable root-associated bacterial populations in maize seedlings under salt stress.

Treatment	Total culturable root-associated bacterial population (CFU g^−1^ fresh root)
S	(3.2±1.5) × 10^2b^
S+R	(1.7±0.8) × 10^4a^
S+R+MT	(2.1±0.7) × 10^4a^
