# Supplementary material for: Combined Bacillus velezensis–Brevibacterium frigoritolerans Biostimulant and Melatonin Improves Salt Tolerance in Maize Seedlings and Is associated with Physiological and Transcriptome–Metabolome Changes
Source: J Microbiol Biotechnol. 2026 Oct 13;36:e2606019. doi: 10.4014/jmb.2606.06019 (PMC13725148; doi:10.4014/jmb.2606.06019)
Supplement: Supplementary file 1 [file jmb-36-e2606019-supple.pdf]

**J. Microbiol. Biotechnol. 2026. 36: e2606019**

https://doi.org/10.4014/jmb.2606.06019

# Supplementary Figures and Tables

## Combined *Bacillus velezensis* – *Brevibacterium frigoritolerans* Biostimulant and Melatonin Improves Salt Tolerance in Maize Seedlings and Is Associated with Physiological and Transcriptome – Metabolome Changes

Xiaoyong Li, Chen Wang*, Qing Feng*, Yu Qiao, and Yuning Zhang

*College of Environmental Science & Engineering, Qilu University of Technology* (*Shandong Academy of Sciences*)*, Jinan, Shandong 250353, P. R China*

*Corresponding author : shanqing123@126.com (Chen Wang), qingfeng@qlu.edu.cn (Qing Feng)

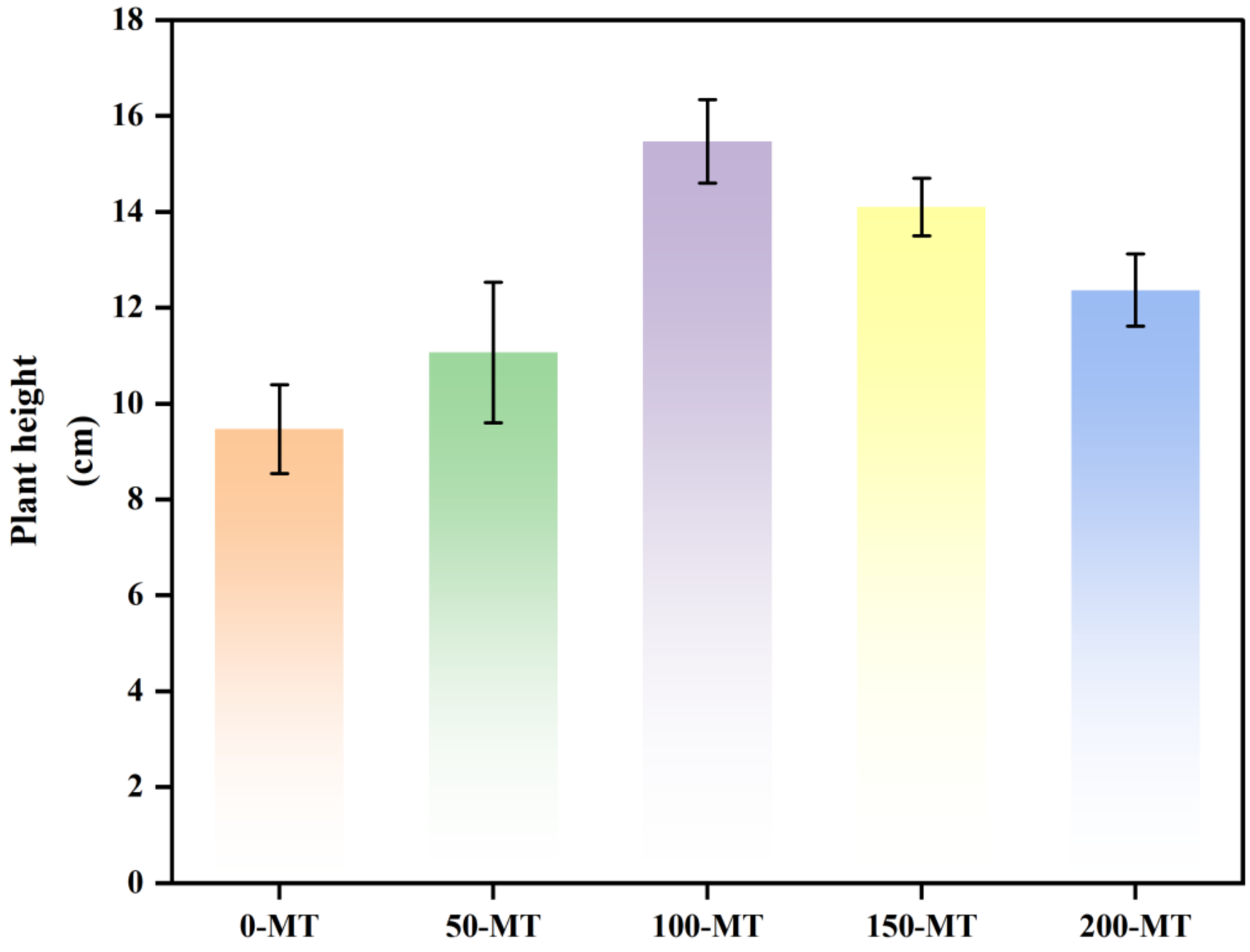


**Fig. S1. Effects of different melatonin (MT) concentrations on plant height of maize seedlings under salt stress conditions**. Data are presented as means ± SD of three biological replicates (n = 3).

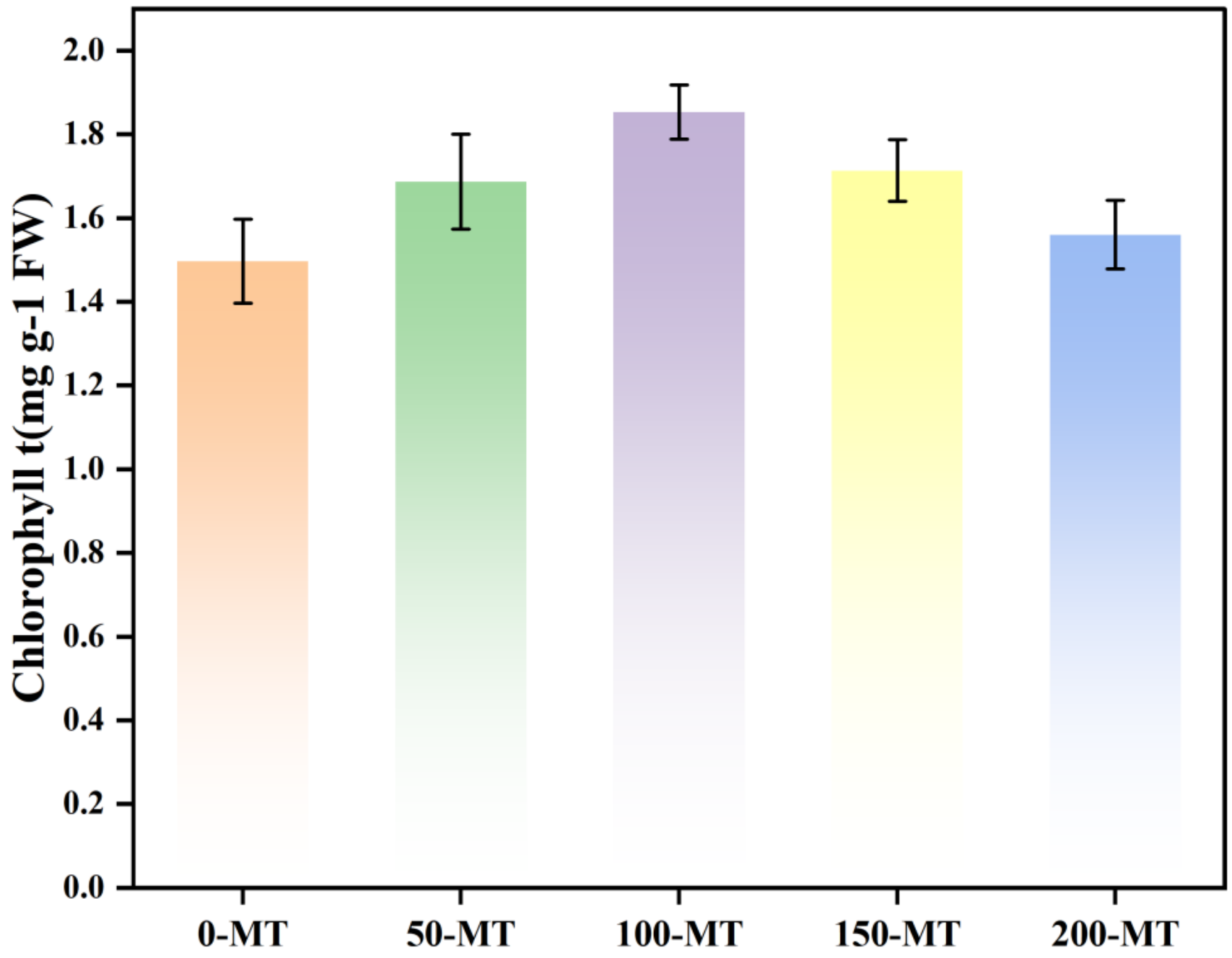


**Fig. S2. Effects of different melatonin (MT) concentrations on chlorophyll content in maize seedlings under salt stress conditions**. Data are presented as means ± SD of three biological replicates (n = 3).

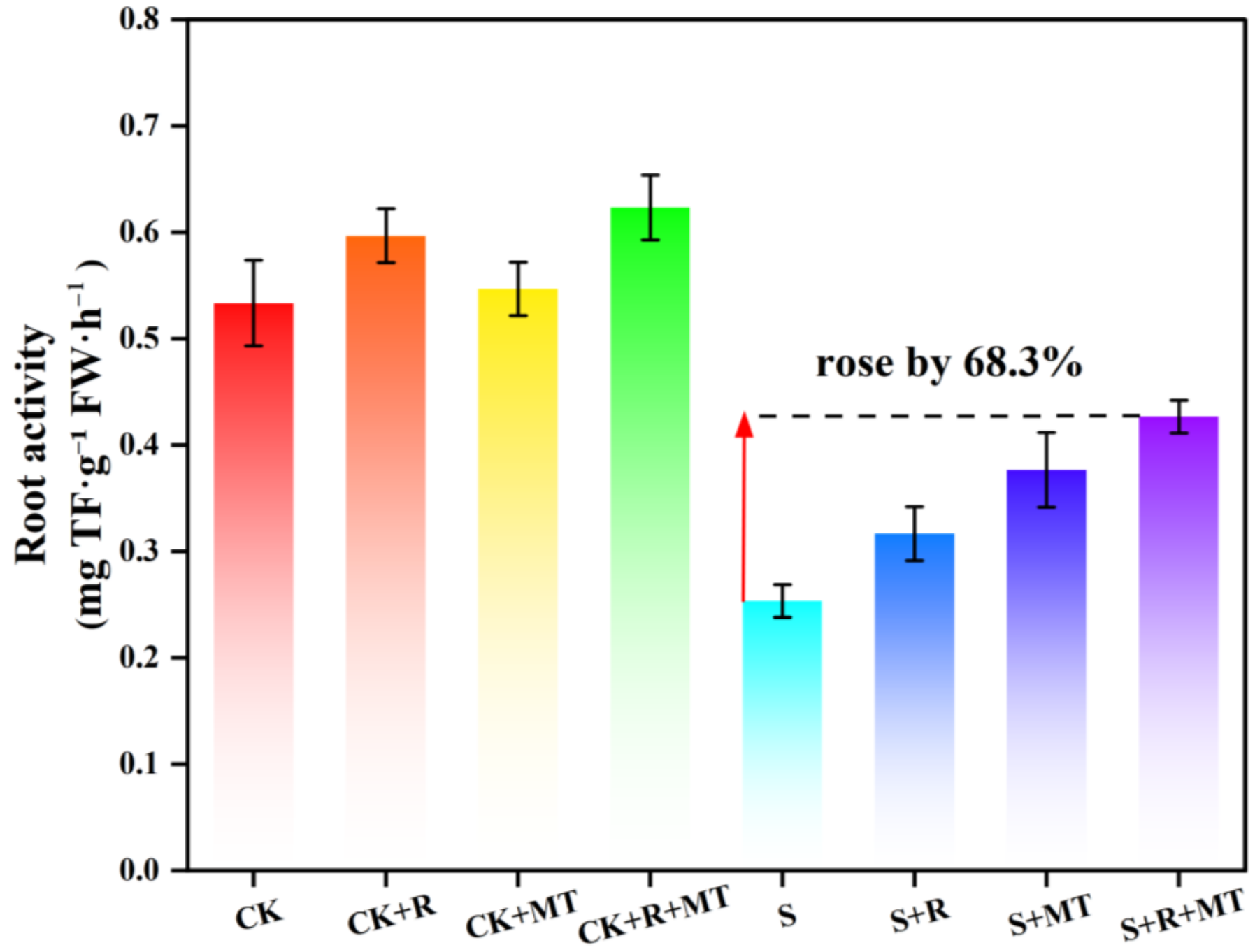


**Fig. S3. Effects of PGPR inoculation, exogenous melatonin (MT), and their combined treatment on root activity in maize seedlings under control and salt stress conditions**. Data are presented as means ± SD of three biological replicates (n = 3).

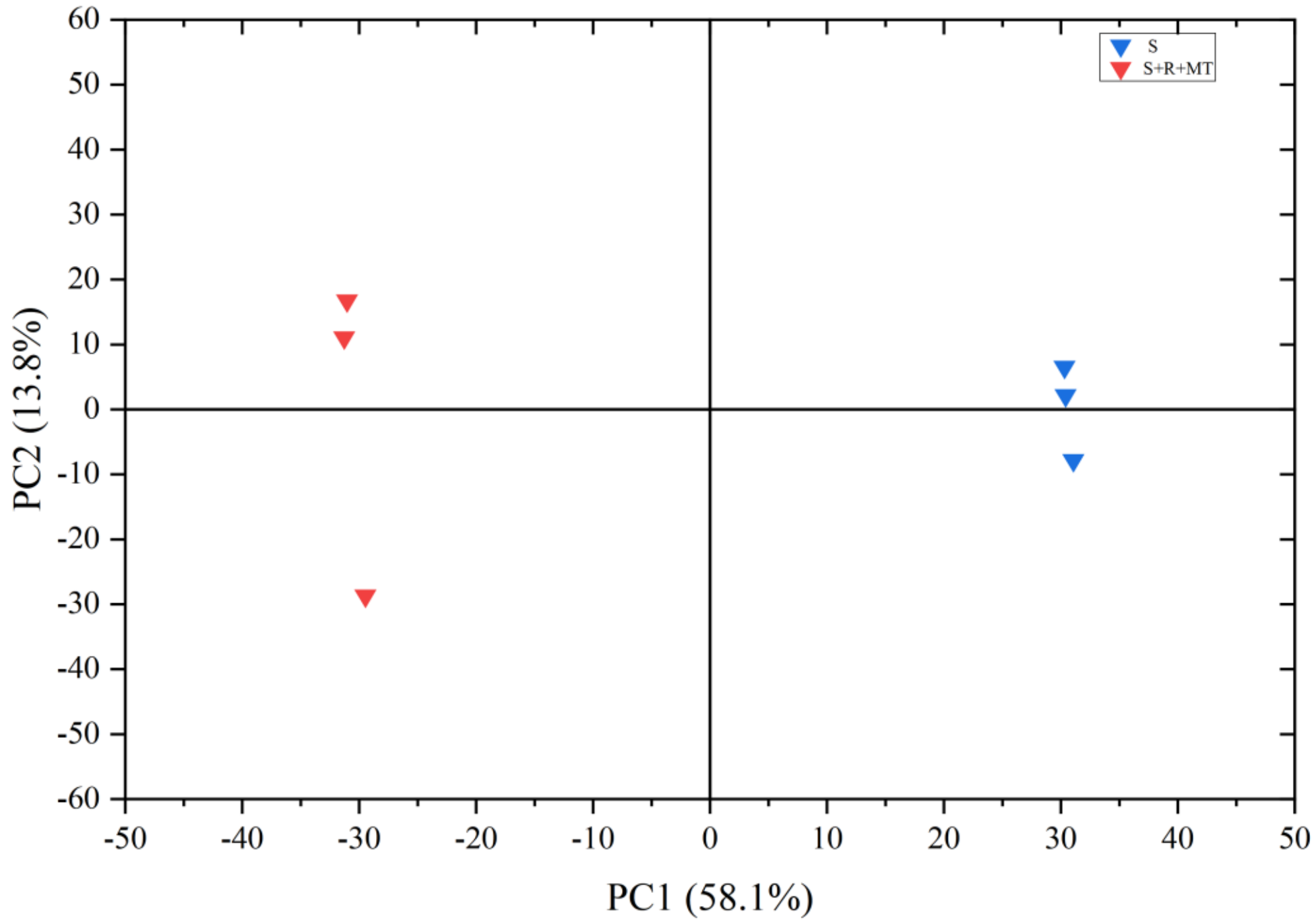


**Fig. S4. Principal component analysis (PCA) based on all differential metabolites identified between the S and S+R+MT groups.**

Each point represents one biological replicate.

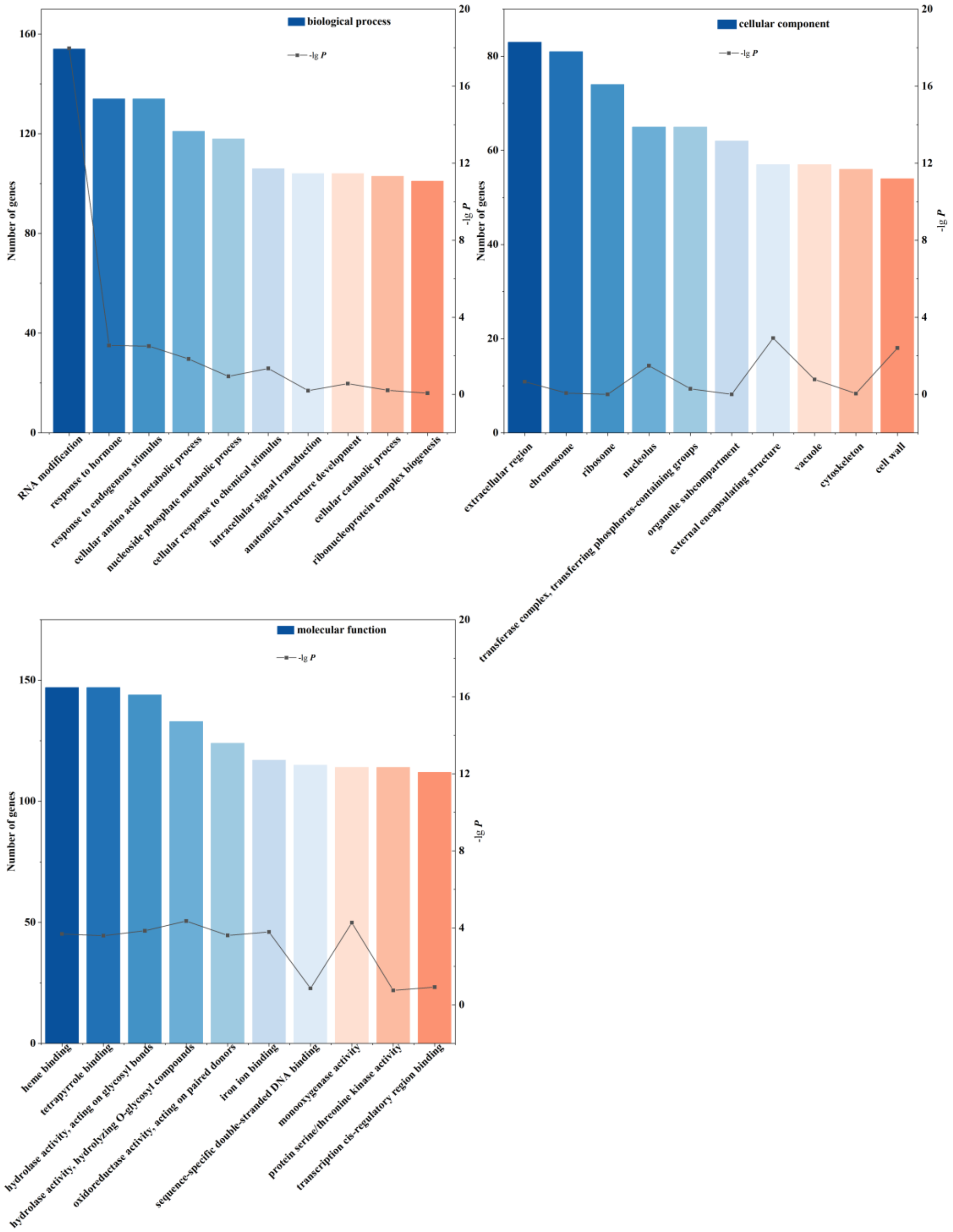


**Fig. S5.** Gene Ontology (GO) enrichment analysis of differentially expressed genes (DEGs) between the S and S+R+MT groups. The enriched GO terms are shown in the categories of biological process, cellular component, and molecular function. Bar height represents the number of genes enriched in each term, and the line indicates the enrichment significance level as −lg P.

**Table S1. RNA-seq sequencing quality statistics.**

| Sample | Raw reads | Clean reads | Clean reads ratio (%) | Q30 (%) | Mapping rate (%) |
|---|---|---|---|---|---|
| S3_3_1 | 67,544,678 | 67,524,480 | 100.0 | 97.1 | 92.27 |
| S3_3_2 | 65,366,332 | 65,354,844 | 100.0 | 96.5 | 93.72 |
| S3_3_3 | 47,136,700 | 47,118,276 | 100.0 | 96.9 | 93.14 |
| S_R_MT1_3_1 | 59,816,118 | 59,789,222 | 100.0 | 97.2 | 86.89 |
| S_R_MT1_3_2 | 63,418,262 | 63,410,114 | 100.0 | 97.0 | 93.14 |
| S_R_MT1_3_3 | 68,911,428 | 68,899,918 | 100.0 | 96.9 | 93.58 |

Note: Q30 indicates the percentage of bases with a Phred quality score ≥30. Mapping rate represents the percentage of clean reads aligned to the reference genome. The average mapping rate across all transcriptome libraries was 92.96%.

**Table S2. Three-way ANOVA analysis of physiological and biochemical traits.**

| Trait | Salt | PGPR | MT | Salt × PGPR | Salt × MT | PGPR × MT | Salt × PGPR × MT |
|---|---|---|---|---|---|---|---|
| Plant height | *** | ** | *** | ** | *** | ns | ns |
| Root length | *** | * | ** | ns | ns | ns | ns |
| Fresh weight | *** | ** | *** | ns | ** | ns | ns |
| Dry weight | *** | *** | *** | ns | * | ns | ns |
| Root activity | *** | *** | *** | ns | *** | ns | ns |
| Chlorophyll a | *** | *** | *** | ns | *** | ns | * |
| Chlorophyll b | *** | *** | *** | ns | ns | ns | ns |
| Total chlorophyll | *** | *** | *** | ns | *** | ns | ns |
| Carotenoids | *** | *** | *** | * | *** | ns | ns |
| Proline | *** | *** | *** | ** | ns | ns | ns |
| Soluble sugar | *** | *** | *** | ns | *** | ns | ns |
| MDA | *** | *** | *** | ** | * | ns | ns |
| $H_2O_2$ | *** | *** | *** | * | *** | ns | ns |
| POD | *** | ** | *** | ns | *** | ns | ns |
| SOD | *** | ns | *** | ns | * | ** | ns |
| CAT | *** | *** | *** | ns | *** | ns | ** |
| APX | *** | *** | *** | *** | ns | ns | ns |

Note: ns indicates no significant difference; *, **, and *** indicate significance at $P < 0.05$, $P < 0.01$, and $P < 0.001$, respectively.
